# A continuously efficient O_2_-supplying strategy for long-term modulation of hypoxic tumor microenvironment to enhance long-acting radionuclides internal therapy

**DOI:** 10.1186/s12951-023-02268-5

**Published:** 2024-01-03

**Authors:** Jingchao Li, Tingting Wang, Yuanfei Shi, Zichen Ye, Xun Zhang, Jiang Ming, Yafei Zhang, Xinyan Hu, Yun Li, Dongsheng Zhang, Qianhe Xu, Jun Yang, Xiaolan Chen, Nian Liu, Xinhui Su

**Affiliations:** 1 Department of Nuclear Medicine, The First Affiliated Hospital, Zhejiang University School of Medicine, Hangzhou, 310003 China; 2 State Key Laboratory for Physical Chemistry of Solid Surfaces, Collaborative Innovation Center of Chemistry for Energy Materials, and Engineering Research Center for Nano-Preparation Technology of Fujian Province, College of Chemistry and Chemical Engineering, Xiamen University, Xiamen, 361005 China; 3 Department of Hematology, The First Affiliated Hospital, Zhejiang University School of Medicine, Hangzhou, 310003 China; 4 State Key Laboratory of Molecular Vaccinology and Molecular Diagnostics & Center for Molecular Imaging and Translational Medicine, School of Public Health, Xiamen University, Xiamen, 361005 China

**Keywords:** Radionuclides internal therapy, Pd-based nanomaterials, Continuous hypoxia relief, Moderate NIR-II photothermal therapy

## Abstract

Radionuclides internal radiotherapy (RIT) is a clinically powerful method for cancer treatment, but still poses unsatisfactory therapeutic outcomes due to the hypoxic characteristic of tumor microenvironment (TME). Catalase (CAT) or CAT-like nanomaterials can be used to enzymatically decompose TME endogenous H_2_O_2_ to boost TME oxygenation and thus alleviate the hypoxic level within tumors, but their effectiveness is still hindered by the short-lasting of hypoxia relief owing to their poor stability or degradability, thereby failing to match the long therapeutic duration of RIT. Herein, we proposed an innovative strategy of using facet-dependent CAT-like Pd-based two-dimensional (2D) nanoplatforms to continuously enhance RIT. Specifically, rationally designed 2D Pd@Au nanosheets (NSs) enable consistent enzymatic conversion of endogenous H_2_O_2_ into O_2_ to overcome hypoxia-induced RIT resistance. Furthermore, partially coated Au layer afford NIR-II responsiveness and moderate photothermal treatment that augmenting their enzymatic functionality. This approach with dual-effect paves the way for reshaping TME and consequently facilitating the brachytherapy ablation of cancer. Our work offers a significant advancement in the integration of catalytic nanomedicine and nuclear medicine, with the overarching goal of amplifying the clinical benefits of RIT-treated patients.

**Graphical abstract:**

**Figure:**
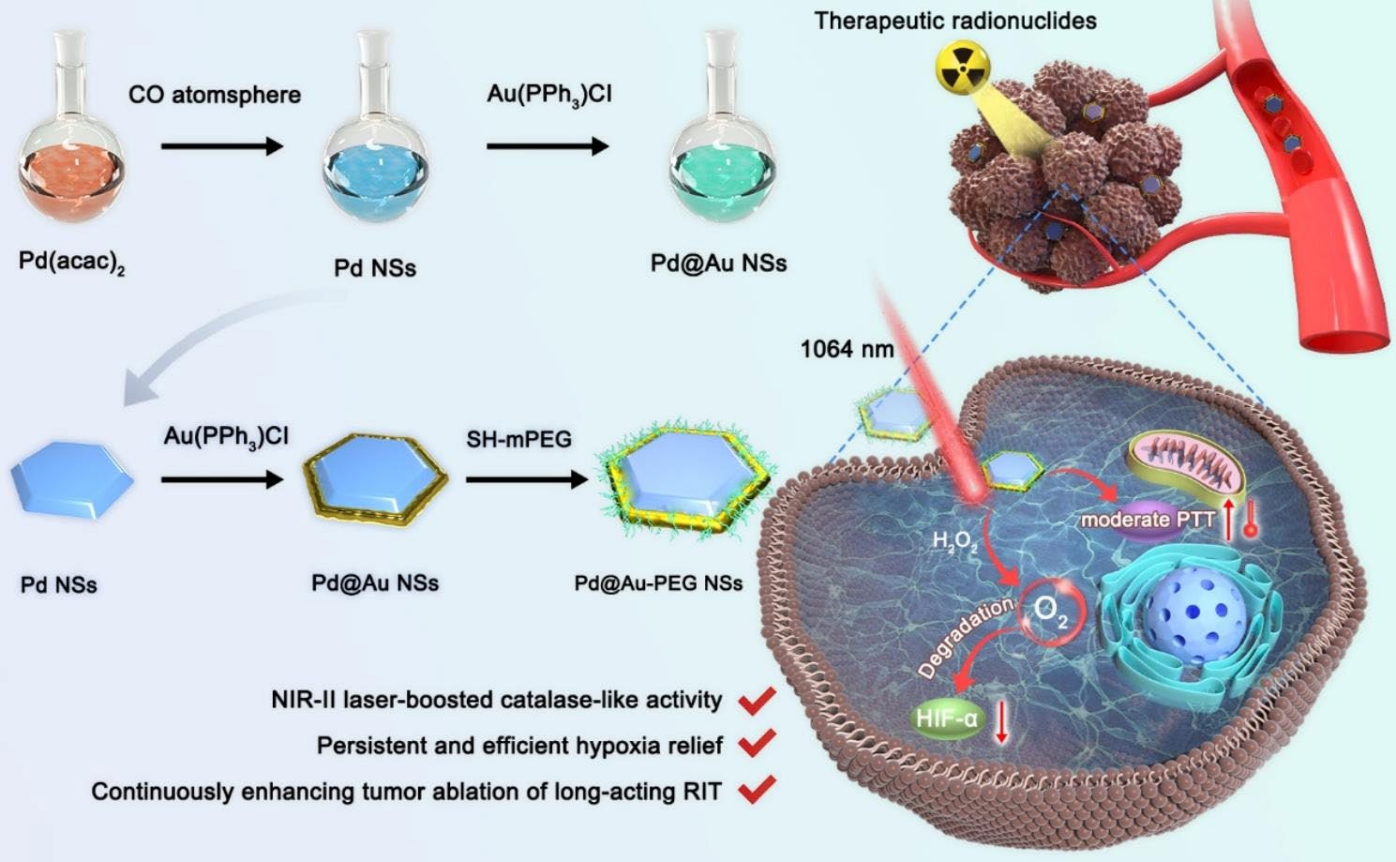

**Supplementary Information:**

The online version contains supplementary material available at 10.1186/s12951-023-02268-5.

## Introduction

Radionuclides internal radiotherapy (RIT) stand out as a pivotal facet of precision medicine owing to its high tumor-specificity and remarkable therapeutic efficiency [1]. For example, lutetium-177(^177^Lu)-, iodine-131(^131^I)-, Yttrium-90 (^90^Y)- or Actinium-225 (^225^Ac)-labeled agents have garnered increasing attention in both pre-clinical studies and clinical translation trials [2–4]. After being precisely delivered into tumors via targeting motifs (including peptides, antibodies, nanomaterials, etc.), these therapeutic radionuclides can induce cell apoptosis through localized high-energy ionizing radiation-mediated double-stranded DNA damage [5, 6]. Often the tumoricidal effect of RIT is not only dependent on the injected radionuclides dose, but also affected by some specific features of tumor microenvironment (TME), such as hypoxia. Hypoxia, as the result of rapid and uncontrollable proliferation of malignant cancer cells, can cause inefficient generation of reactive oxygen species (ROS) and post-treatment repair of DNA damage, thereby contributing to the resistance of cancers towards photodynamic therapy, chemotherapy and RIT [7–10]. Hypoxia also enables the up-regulation of multiple protective signals or growth factors of cancer cells, thus reducing the tumor-suppressing effect of RIT [11–14]. Therefore, efficiently overcoming hypoxia-induced tumor radio-resistance plays a vital role in elevating the therapeutic index of clinically available radiopharmaceuticals.

Nanobiotechnology based hypoxia-alleviating methods have been utilized to weaken TME hypoxia, such as physically enhancing O_2_ delivery via O_2_-loading vehicles, and utilizing catalase (CAT) or CAT-mimicking nanomaterials for in situ chemically enzyme-catalyzed O_2_ generation [15–20]. CAT-mimicking nanomaterials adequately leverage the over-produced H_2_O_2_ in TME as the O_2_ donor, thus theoretically displaying high tumor specificity and hypoxia-alleviating capability during the treatment of RIT [21]. For example, catalase albumin-templated MnO_2_ nanoparticles (NPs) with CAT-like activity enable the decomposition of endogenous H_2_O_2_ to produce O_2_ for hypoxia management and ^131^I-involved RIT enhancement [22]. However, most of these enzyme-mimicking nanomaterials cannot supply a continual generation of O_2_ for enhanced RIT due to their intrinsic degradability and poor stability in harsh TME [23]. Palladium (Pd)-based two-dimensional (2D) nanomaterials with multiplex enzyme-like activity exhibited tremendous potential in nanotheranosctics due to their large surface area for drug loading, high photothermal-converting efficiency, as well excellent passive tumor-targeting ability and biosafety [24–26]. Importantly, on the account of the stable exposure of Pd (111) crystal facet, 2D Pd-based nanosheets (NSs) with robust CAT-like activity can provide long-term hypoxia relief to strengthen O_2_-dependent tumor therapy, offering a potentially ingenious approach to address the duration mismatching between hypoxia relief and RIT [27–30]. Furthermore, the photothermal property of Pd-based nanomaterials not only does good to hyperthermia treatment of cancerous cells [31], but also physiologically conquers hypoxic tumor TME through enhancing vessel dilation and blood perfusion via moderate photothermal treatment (PTT) [32, 33].

Herein, we pioneered the first exploration of using Pd-based nanostrucrtures (Pd@Au NSs) to boost tumor-ablating efficiency of clinical RIT formulation (^90^Y resin spheres in this work) (Scheme 1). Polyethylene glycol (PEG)-modified Pd@Au NSs exhibited robust CAT-like activity and second near-infrared (NIR-II) photothermal performance. And these Pd@Au-PEG NSs were able to efficient accumulated in tumor by enhanced permeability and retention (EPR) effect to promote sustained hypoxia alleviation, which could match with the long-term therapeutic duration of internal ^90^Y radionuclides. Through the synergistic guidance of living imaging, a controlled NIR-II PTT regimen was implemented to not only enhance the enzyme-mimicking performance of Pd@Au-PEG NSs [34], but also induce vessel dilation and improve blood perfusion to modulate hypoxic TME. Upon the brachytherapy of ^90^Y resin spheres, Pd@Au-PEG NSs along with low-power density NIR-II laser irradiation enabled the thorough ablation of subcutaneous 4T1 tumors, well demonstrating the feasibility and efficacy of this RIT-sensitizing strategy.

## Results and discussion

### Synthesis and characterizations of Pd@Au-PEG NSs

**Fig. 1:**
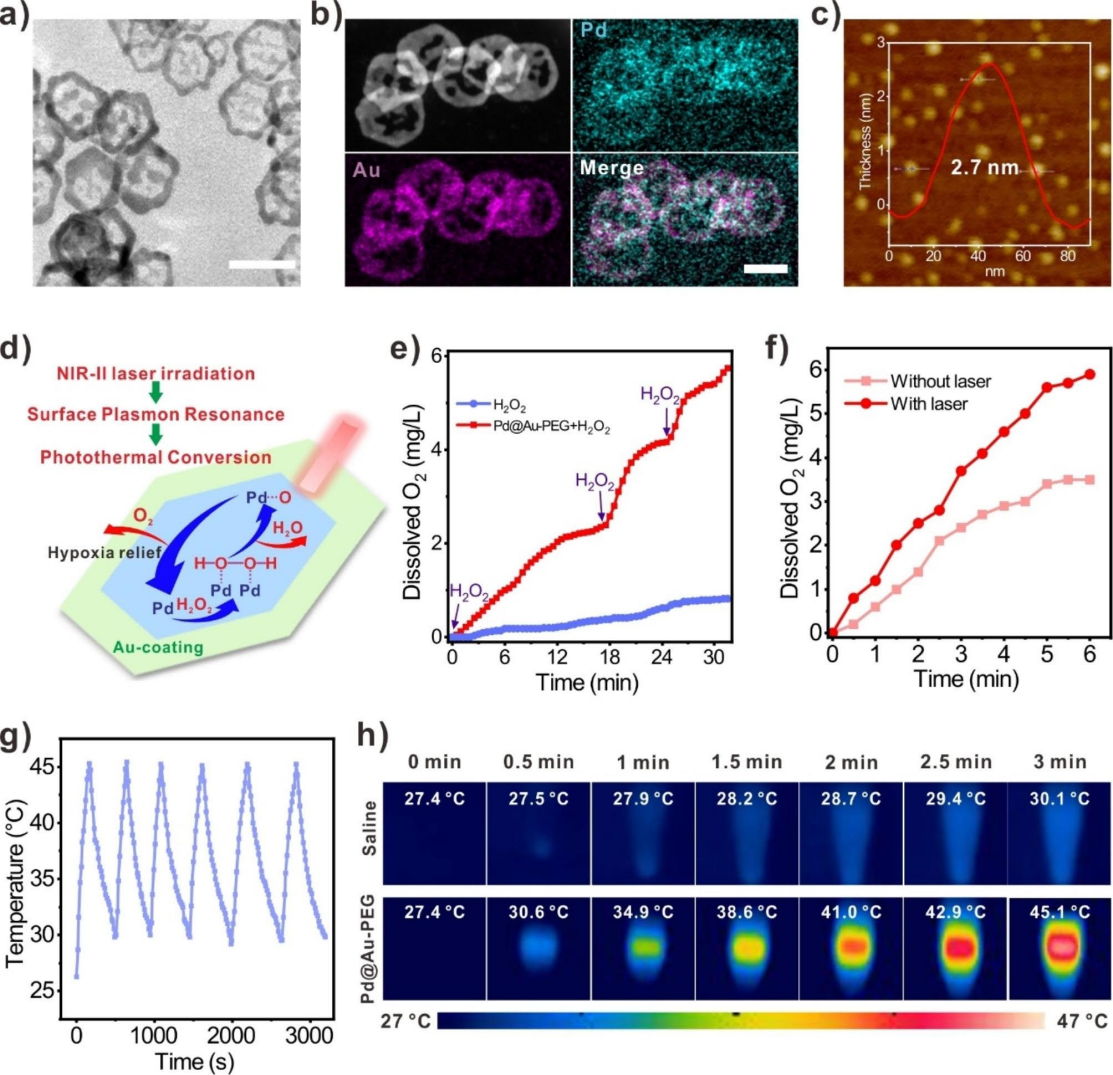
Characterizations, CAT-like activity and NIR-II photothermal property of Pd@Au-PEG NSs. (**a**) TEM image of Pd@Au-PEG NSs (Scale bar = 50 nm). (**b**) Element mapping analysis of Pd@Au-PEG NSs (Scale bar = 20 nm). (**c**) AFM image and the thickness measurement of Pd@Au-PEG NSs. (**e**) The UV-Vis-NIR absorption spectrum of Pd@Au-PEG NSs. (**d**) Schematic diagram of mechanism regarding the enzymatic activity and photothermal responsiveness of Pd@Au-PEG NSs. (**e**) CAT-like activity of Pd@Au-PEG NSs and their property of continuously decomposing H_2_O_2_ for O_2_ evolution with repeated H_2_O_2_ addition. (**f**) NIR-II surface plasmon resonance-boosted enzymatic activity of Pd@Au-PEG NSs. (**g**) Photothermal stability of Pd@Au-PEG NSs. (**h**) Infrared thermal images of saline (control group) and Pd@Au-PEG NSs solution under the excitation of NIR-II laser (1064 nm, 0.3 W cm^− 2^)

To construct a 2D NIR-II-responsive Pd-based nanoplatforms with CAT-like activity, the hexagonal Pd@Au-PEG NSs with the homogeneous size of 30 nm were fabricated through the simple “seeds-surface growth” preparation approach. The reason of choosing 30 nm Pd NSs as the seeds among candidates was mainly because they can exhibit the highest tumor accumulation by EPR effect [35]. Then, PEG modification was carried out to improve the dispersion and biocompatibility of Pd@Au NSs. Transmission electron microscopy (TEM) images showed the hexagonal morphology and incomplete Au layer of Pd@Au-PEG NSs with ~ 30 nm of size (diagonal length) (Fig. 1a; Additional file 1: Fig. S1). Element mapping analysis further corroborated the presence of both Pd and Au within the fabricated nanoarchitectures (Fig. 1b). Inductively coupled plasma mass spectrometry (ICP-MS) analysis quantitatively indicated the contents of Au and Pd were 78.7 wt% and 21.3 wt%, respectively (Additional file 1: Fig. S2). Atomic force microscopy (AFM) was employed to determine the thickness of Pd@Au NSs, which was measured with 2.7 nm, suggesting the characteristic 2D structural nature of Pd@Au-PEG NSs (Fig. 1c).

The absorption spectrum of Pd@Au-PEG NSs showed a strong NIR-II responsiveness, more redshifted than the precursor Pd NSs because of Au coating. (Additional file 1: Fig. S3). The PEG modification led to a distinct decrease in the surface potential of Pd@Au NSs, transitioning from a positive value (13.93 ± 0.66 mV due to the triphenylphosphine on the surface of Au) to a moderately negative value (-5.41 ± 1.08 mV) (Additional file 1: Fig. S4). To test their stability, Pd@Au-PEG NSs were subjected to the incubation in saline over intervals of 1, 3, 5, and 7 days, and their hydrodynamic sizes and absorption spectrum were recorded. The hydrodynamic size of Pd@Au-PEG NSs demonstrated exceptional stability over the prolonged incubation periods, which is mainly attributed to the surface PEG modifacation (Additional file 1: Fig. S5). Additionally, a week incubation in the saline would not affect the inherent NIR-II absorption of Pd@Au-PEG NSs (Additional file 1: Fig. S6).

### CAT-like activity and NIR-II photothermal conversion performance of Pd@Au-PEG NSs

In the part of nanomaterials designing, we planned to construct a Pd-based nanoplatforms that well retaining the inherent CAT activity of Pd NSs and possessing NIR-II responsiveness *via* finely tuning the Au coating areas on the surface of Pd NSs, realizing the combination of enzymatic O_2_ evolution and moderate photothermal stimulation-induced tumor blood perfusion enhancement for synergetic hypoxia relief (Fig. 1d). Hence, the CAT-like activity of Pd@Au-PEG NSs was firstly assessed by detecting the O_2_ concentration variation in the co-incubation of Pd@Au-PEG NSs and H_2_O_2_. As shown in Fig. 1e, H_2_O_2_ alone failed to efficiently produce O_2_ without the addition of enzyme, while the co-incubated solution containing Pd@Au-PEG NSs and H_2_O_2_ displayed obvious O_2_ generation, confirming the CAT-like activity and hypoxia-relieving ability of Pd@Au-PEG NSs. However, as we mentioned above, transitory hypoxia modulation cannot match the ^90^Y resin spheres-based RIT in the terms of duration. Given the dynamic supplement of H_2_O_2_ in TME, we repeatedly added H_2_O_2_ with the same amount when the previously added H_2_O_2_ was completely consumed, and continuously monitored the dissolved O_2_ concentration. Profiting from their stable CAT-like activity, Pd@Au-PEG NSs enabled persistent H_2_O_2_ decomposition for repeatable O_2_ evolution (Fig. 1e), thus holding a great promise for long-term hypoxia relief. To explore whether NIR-II laser stimulation can further enhance the enzymatic activity of Pd@Au-PEG NSs, the experimental sample was irradiated with a 1064 nm laser (0.3 W cm^− 2^), while the control group was not irradiated by laser. The rate of increase of dissolved O_2_ concentration in two samples indicated the improved CAT-like activity of Pd@Au-PEG NSs upon laser irradiation (Fig. 1f). Based on the result that the solution temperature kept almost unchanged during the laser irradiation, it was rational to propose that the reinforced enzyme-mimicking activity of Pd@Au-PEG NSs was not derived from the photothermal effect, but an enhanced catalysis induced by NIR-II surface plasmon resonance effect. Considering the low pH characteristic of TME, the CAT-like activities of Pd@Au-PEG NSs incubated in the buffer solution with different pH values were further investigated to show the actual performance of Pd@Au-PEG NSs in a TME-mimicking environment. Surprisingly, Pd@Au-PEG NSs could exhibit excellent resistance to the acidic condition, significantly maintaining their CAT-like activity even at the condition of pH 3.5 (87.2% ± 4.3%, taking pH = 7 as the 100%) (Additional file 1: Fig. S7). Therefore, we believe that Pd@Au-PEG NSs can serve as an efficient intratumoral O_2_-generator to reshape a RIT-tailored TME, especially with the assistance of NIR-II laser excitation.

Although the NIR-II responsiveness of Pd@Au-PEG NSs has been clarified by their absorption spectrum, the NIR-II photothermal conversion ability of Pd@Au-PEG NSs still deserves exploration to realize moderate NIR-II PTT. Compared to the hyperthermia treatment that pursuing highly efficient light-to-heat conversion, moderate PTT can decrease the risk of inducing thermal injury to normal tissues around tumor and hold the promise to increase the compliance of patients. Its efficacy more focus on regulating TME or cell signaling to potentiate therapeutic outcomes instead of directly inducing cells death [36–38]. Due to the difficulty to provide hyperthermia to kill cancer cells, moderate PTT becomes a fresh path to utilize heat for enhanced tumor combined therapy, especially in clinical situations. To pioneer the synergy of RIT and moderate PTT, the NIR-II photothermal conversion performance of Pd@Au-PEG NSs with various concentrations were studied. Both the concentration of Pd@Au-PEG NSs and the NIR-II laser (1064 nm) power density had vital effect on the photothermal converting outcomes (Additional file 1: Fig. S8). Furthermore, the stability of photothermal converting property of Pd@Au-PEG NSs was unravelled using the cyclic “heating-cooling” experiments for 6 times (Fig. 1g). The morphologies of Pd@Au-PEG NSs before and after photothermal stability test were almost the same (Additional file 1: Fig. S9). These results also set up a solid ground to leverage multi-times moderate PTT for achieving repeatable augment of enzymatic activity and tumor blood perfusion. Under the condition of 0.3 W cm^− 2^ laser stimulation, Pd@Au-PEG NSs solution (40 ppm) would reach 45.1 ℃ within 3 min, while the temperature of saline control group was only increased from 27.4 ℃ to 30.1 ℃ (Fig. 1h), revealing the excellent NIR-II photothermal efficacy of Pd@Au-PEG NSs and the appropriateness of adopting 0.3 W cm^− 2^ as the laser power density for operating in vivo NIR-II moderate PTT, which could potentially keep mild speed of temperature increase and reduce the high-energy laser damage towards skin.

### Subcellular localization of Pd@Au-PEG NSs and in vitro enhanced RIT

**Fig. 2:**
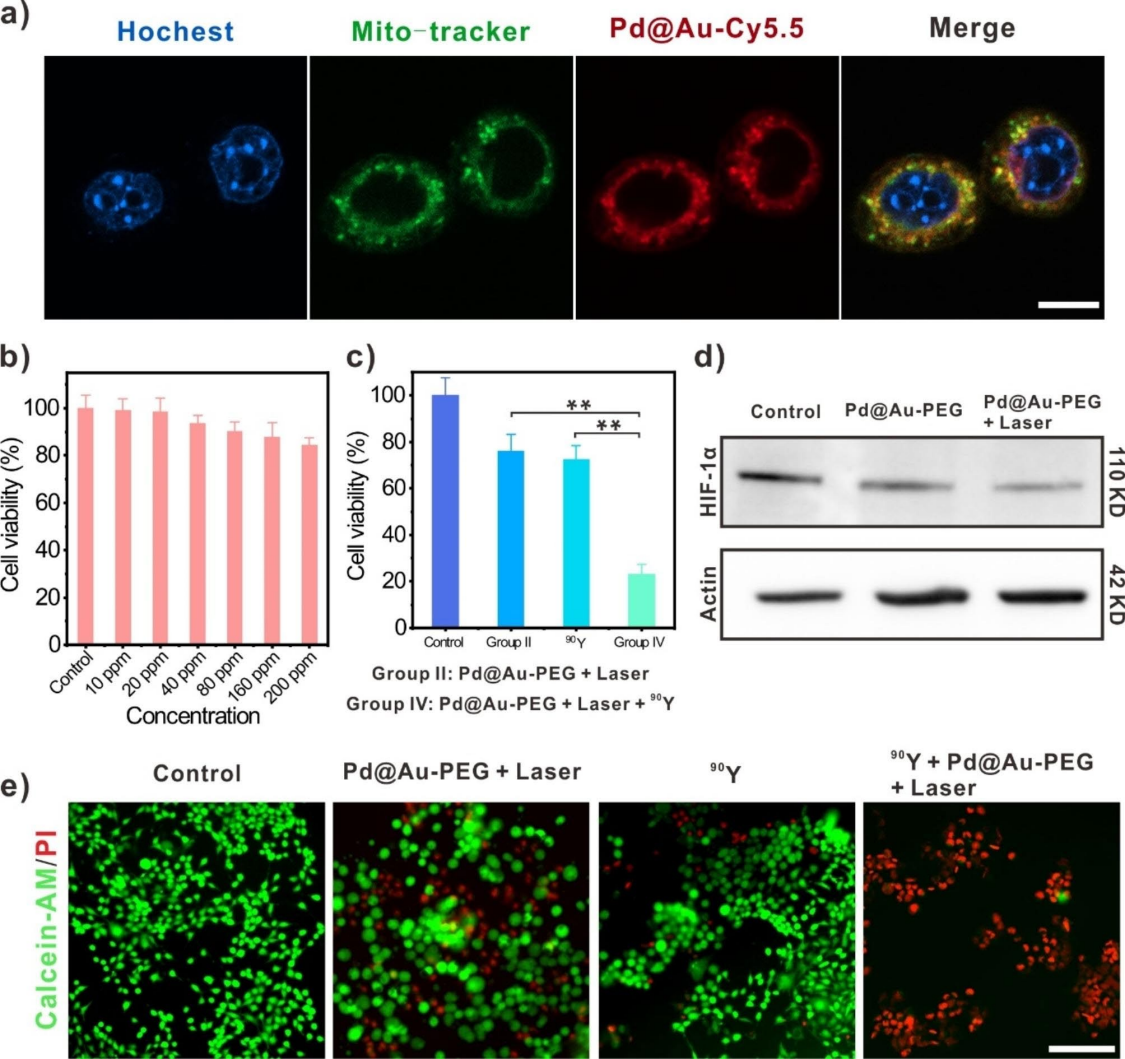
Cell assays. (**a**) Confocal fluorescence microscope images of 4T1 cancer cells treated by Hochest (blue fluorescence), Mito-tracker (green fluorescence) and Pd@Au-Cy5.5 (red fluorescence) to demonstrate their mitochondria-targeting ability (Scale bar: 10 μm). (**b**) The cell-level biocompatibility of Pd@Au-PEG NSs. (**c**) Cells viabilities of 4T1 cancer cells after different treatments for 24 h. (**d**) Western blotting images of HIF-1α expression of 4T1 cancer cells after different treatments for 24 h under the hypoxic culture condition. (**e**) Fluorescence microscopy images of 4T1 cancer cells dual-stained with Calcein-AM (green fluorescence) and PI (red fluorescence) after different treatments for 24 h (Scale bar: 50 μm)

Despite great advancements have been witnessed in the tumor theranostics application of 2D Pd-based nanomaterials, there are few works uncovering the specific subcellular localization of these nanoagents. Inspired by the inherent mitochondria-targeting function of triphenylphosphine [38, 39], we hypothesized that these Pd@Au-PEG NSs with triphenylphosphine attachment can exhibit mitochondria-directed behavior at the subcellular level. To visualize intracellular Pd@Au NSs in confocal fluorescence microscope, Cy5.5-NHS was employed as the fluorescence label to conjugate with the NH_2_-PEG-SH-modified Pd@Au NSs through the formation of stable amide bond to afford Cy5.5-labeled Pd@Au-PEG NSs (denoted by Pd@Au-Cy5.5). After being co-cultured with Pd@Au-Cy5.5 for 3 h, 4T1 cancer cells were treated with Hochest 33342 and Mito-Tracker Green in turn for providing blue fluorescence signal in cell nucleus and green fluorescence signal in mitochondria, respectively. Being consistent with our hypothesis, the red fluorescence signal emitted by Pd@Au-Cy5.5 displayed remarkable overlap with the Mito-tracker-resulted green fluorescence area (Pearson’s correlation coefficients = 0.91 ± 0.01) (Fig. 2a), while the Pd-Cy5.5 failed to specifically target mitochondria (Pearson’s correlation coefficients = 0.47 ± 0.02) (Additional file 1: Fig. S10), indicating that Pd@Au-PEG NSs can play the role of subcellular-targeting carrier for mitochondria-associated biomedical applications. Meanwhile, the evaluation on cytocompatibility of Pd@Au-PEG NSs confirmed their biosafety of these CAT-like nanomaterials at the given incubation dose (Fig. 2b).

Subsequently, the cell proliferation-inhibiting effect and mechanism of this enhanced RIT method was also explored using standard CCK8 assay and western blotting analysis. Given the feature of TME with high level H_2_O_2_ (50–100 μM), all the 4T1 cell groups were cultured with the medium containing 100 μM H_2_O_2_ (the enzymatic substrate of Pd@Au-PEG NSs) under the hypoxic condition. Compared with the group treated by ^90^Y alone or Pd@Au-PEG NSs + laser, the co-treatment of ^90^Y and photo-enhanced Pd@Au-PEG NSs showed significantly higher efficacy in killing cancer cells (~ 76.9%) (Fig. 2c). This fully shed light on the importance of O_2_ level for augmenting RIT benefits. Then, we evaluated the hypoxia inducible factor-α (HIF-α) expression of 4T1 cells with various treatments. Under the culture condition simulating hypoxic TME, 4T1 cells signed with significant HIF-α expression, which might mechanistically pose the radio-resistance of cancer cells towards RIT. As expected, the HIF-α expression of cells was obviously downregulated in the Pd@Au-PEG NSs co-incubation group, and the HIF-α-downregulating efficiency could be further improved with the aid of NIR-II laser owing to the photo-enhanced enzymatic activity of Pd@Au-PEG NSs (Fig. 2d), demonstrating the necessity for coupling Pd@Au-PEG NSs and NIR-II laser stimulation to boost hypoxia relief and sensitize cancer cells to RIT. To visually display the enhanced therapeutic effect achieved by hypoxia relief, 4T1 cells with various treatments were co-treated with Calcein-AM and propidium iodide (PI) probes, which could stain living cells and dead cells, respectively. Compared with the control group, the combination of Pd@Au-PEG NSs and low-density laser stimulation led to negligible effect on the survival state of cells, and the single-modal ^90^Y treatment also enabled limited cell-killing efficiency. With help of photo-enhanced hypoxia relief, the therapeutic outcome of ^90^Y-mediated RIT was greatly improved, evidenced by the emergence of numerous red fluorescence associated with dead cells (Fig. 2e).

### In vivo monitoring the biodistribution of Pd@Au-PEG NSs and their ability in persistently suppressing HIF-α expression

**Fig. 3:**
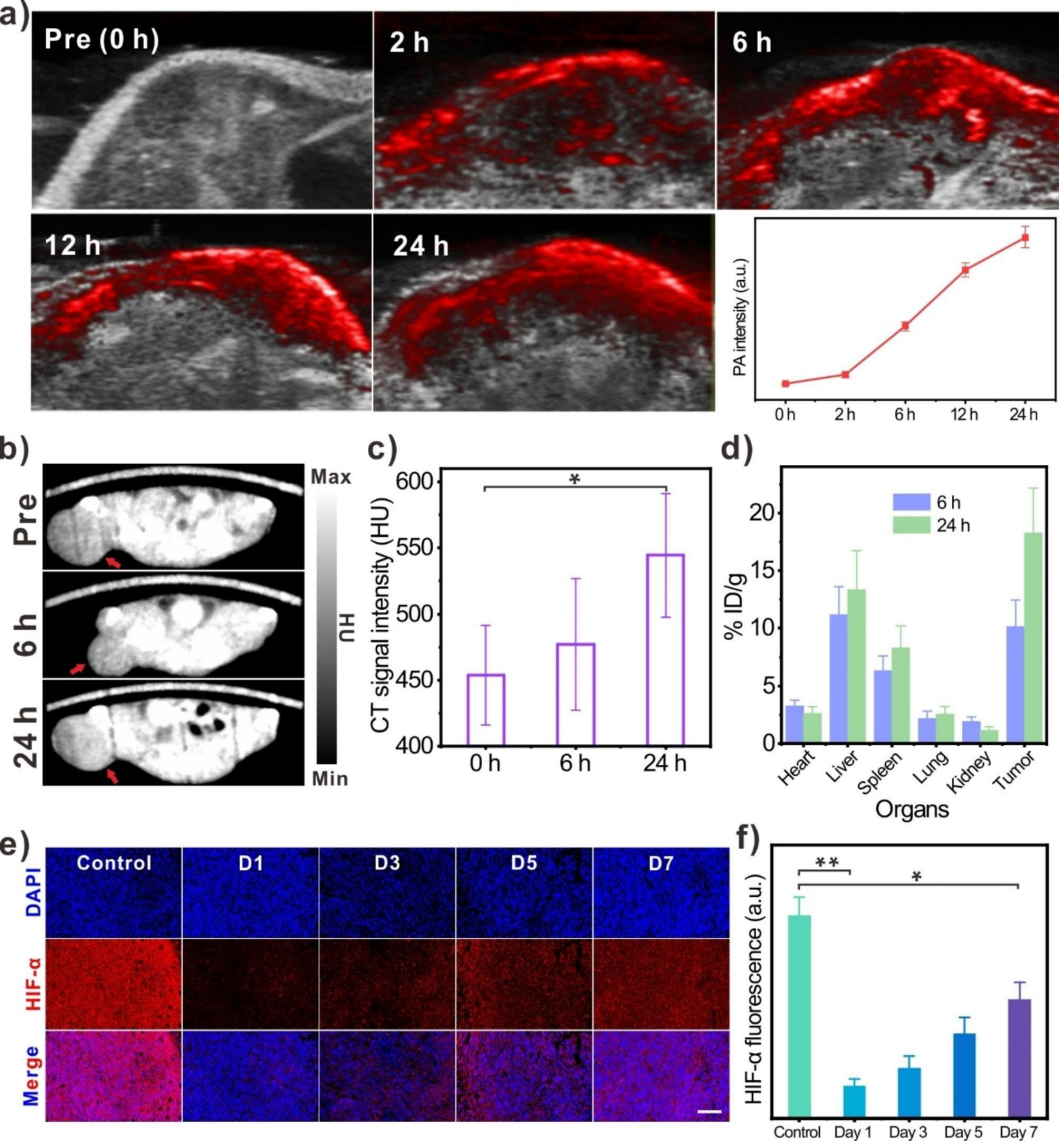
Living imaging and in vivo HIF-α-inhibiting effect of NIR-II-enhanced Pd@Au-PEG NSs. (**a**) PA imaging of Pd@Au-PEG NSs-injected mice (n = 3) and the quantitative PA signal analysis in tumor at various time points (pre, 2 h, 6 h, 12 and 24 h). (**b**) CT imaging of Pd@Au-PEG NSs-injected mice (n = 3) at 6 and 24 h post-injection (Red arrows represent the tumor sites). (**c**) In vivo CT signal in tumor sites post-injection of Pd@Au-PEG NSs. (**d**) In vivo biodistributions of Pd@Au-PEG NSs by ICP-MS detection (n = 5). (**e**) Immunofluorescence staining of HIF-α expressions in tumors of Pd@Au-PEG NSs-injected mice at day-1, -3, -5 and  -7 post-injection (Scale bar = 50 μm). (**f**). The quantitative analysis of HIF-α fluorescence signal intensities

Living imaging-guided therapy is a widely applied strategy for enhancing the precision, spatiotemporal selectivity, and effectiveness of tumor treatments [40–43]. This approach allows for the optimization of therapeutic schedules and mitigates side-effects. Inspired by this paradigm, we conducted on a comprehensive investigation of the in vivo biodistribution of Pd@Au-PEG NSs at different time points using multi-modal living imaging, aiming at determining the opportune time points for NIR-II laser irradiation and intratumoral injection of ^90^Y resin spheres. Taking advantage of the intrinsic contrast capability of Pd@Au-PEG NSs (Additional file 1: Fig. S11), we employed photoacoustic (PA) imaging to focus on the tumor region of mice injected with Pd@Au-PEG NSs to real-time monitor the accumulation Pd@Au-PEG NSs. As illustrated in Fig. 3a, PA signal became evident at the 2 h post-injection, indicating the remarkable tumor-targeting behavior of Pd@Au-PEG NSs facilitated by the EPR effect. As the imaging time-point extended, the intratumoral PA signal exhibited a notable upward trend. This can be attributed to the prolonged in vivo circulation afforded by the surface-modified long-chain PEG molecules attached to Pd@Au-PEG NSs. Consequently, the highest PA signal within the tumor was recorded at the 24 h post-injection. Simultaneously, owing to the Au-coated layer with its high X-ray attenuation coefficient [44], the utility of Pd@Au-PEG NSs as a contrast agent for computed tomograpgy (CT) imaging was tested (Additional file 1: Fig. S12). This feature endowed Pd@Au-PEG NSs with visibility in CT imaging, as well enabling the visualization of their intratumoral accumulation (Fig. 3b). Like the findings from PA imaging, the CT signal of Pd@Au-PEG NSs within the tumor at the 24 h post-injection markedly surpassed that observed at the 6 h post-injection (Fig. 3c). This result further corroborated the identified optimal timing for conducting NIR-II moderate PTT. Mechanistically, the magnitude of temperature elevation induced by PTT is largely contingent on the photothermal agents (PTA) content and excitation laser power. A higher accumulation of PTA in the tumor signifies a lower requirement for laser power to achieve controlled PTT temperature, featuring the advantage of diminishing the risk of tissue damage stemming from high-power density lasers, emphasizing the critical role of PTA accumulation in achieving the desired therapeutic effect while minimizing damage to healthy tissues [45]. To gain qualitative insights into the whole-body distribution of Pd@Au-PEG NSs, living fluorescence imaging using Pd@Au-Cy5.5 was performed on a nude mice model bearing 4T1 tumors. As illustrated, the visualization of tumor accumulation of Pd@Au-PEG NSs at 6 h post-injection was apparent, and relatively lower compared to the enrichment in liver (Additional file 1: Fig. S13). This discrepancy could be attributed to the reticuloendothelial system, primarily composed of the liver and spleen, which is characterized by rapid and active phagocytosis of nanoparticles (Additional file 1: Fig. S14). Over an extended imaging period, a greater influx of Pd@Au-PEG NSs into the solid tumor through blood circulation was observed, leading to an intensified fluorescence signal within the tumor. This heightened signal directly correlates with an increased uptake of Pd@Au-PEG NSs by the tumor, in alignment with the tendencies observed in the living imaging outcomes and in vivo biodistribution investigations (Fig. 3d).

After confirming the tumor-targeting behaviors of Pd@Au-PEG NSs and identifying the optimal time point to execute moderate PTT, the practical utility of intratumorally administered Pd@Au-PEG NSs, coupled with NIR-II laser assistance, for hypoxia relief was investigated. This was accomplished through immunofluorescence analysis of HIF-α expressions within 4T1 tumors. The TME is characterized by hypoxia, leading to elevated HIF-α expression and a diminished therapeutic index [46, 47]. The histological examination outcomes, characterized by strong red fluorescence, distinctly shed light on the abundance of HIF-α protein within the TME of malignant 4T1 tumors without any treatment (Fig. 3e **(control group)**). At day-1 post-injection of Pd@Au-PEG NSs, the tumor region was subjected to NIR-II laser irradiation. This procedure aimed to amplify the enzyme-like activity of accumulated Pd@Au-PEG NSs and enhance intratumoral blood perfusion through controlled PTT, with the goal of collaboratively alleviating hypoxia. Consequently, this intervention led to a substantial reduction in fluorescence signal, indicative of suppressed HIF-α expression (Fig. 3e). Furthermore, the expression levels of HIF-α within the tumor samples were analysed at day-3, -5, and  -7 following Pd@Au-PEG NSs injection (Fig. 3f). This extended analysis served to reflect the longer-term alterations in the TME’s hypoxic state. The acquired results definitively explained the sustained ability of intratumoral Pd@Au-PEG NSs to consistently and enzymatically decompose H_2_O_2_. This outcome substantiates our expectations concerning the in vivo performance of the meticulously designed Pd@Au-PEG NSs, and establishes a solid foundation for subsequent in vivo enhanced RIT.

### In vivo enhanced ^90^Y resin spheres brachytherapy

**Fig. 4:**
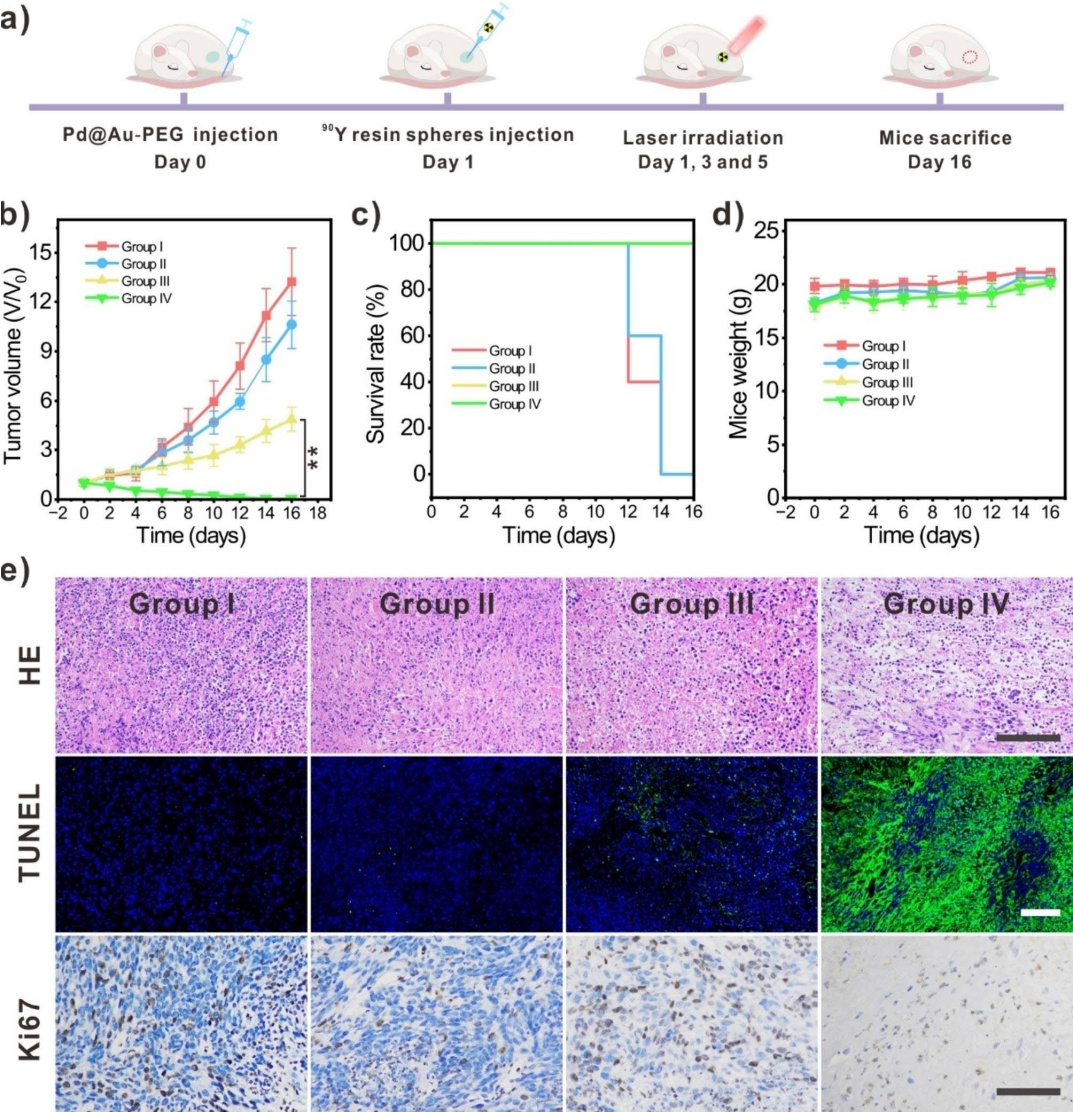
In vivo ^90^Y-resin spheres brachytherapy enhanced by continuous hypoxia relief and NIR-II moderate PTT. (**a**) Schematic diagram of the therapeutic progress. (**b**). The average tumor growth curves of 4T1 subcutaneous tumors with various therapeutics (n = 5). Group I: Saline (control group); Group II: Pd@Au-PEG NSs + laser (moderate PTT); Group III: ^90^Y-micropsheres; Group IV: ^90^Y-micropsheres + Pd@Au-PEG NSs + laser. (**c**) The survival curves of tumor-bearing mice in each treatment group. (**d**) The weight curves of mice in each treatment group. (**e**) H&E staining, TUNEL assay and Ki67 staining of tumor samples from mice in four treatment groups (Scale bar = 50 μm)

Building upon the comprehensive investigations encompassing in vitro activity assessments and in vivo multimodal imaging, the antitumor effect of in vivo RIT involving ^90^Y resin spheres (a clinically-approved radiopharmaceuticals) [48, 49], augmented by multi-faceted hypoxia alleviation, was evaluated in 4T1 subcutaneous tumor models (Fig. 4a). After 7 days post-cancer cells inoculation, the tumors had grown to approximately 80 mm^3^, reaching an appropriate size for the brachytherapy experiments. Subsequently, intravenous administration of Pd@Au-PEG NSs was carried out, followed by the intratumoral injection of ^90^Y resin spheres. Then, multiple NIR-II laser stimulations were executed to simultaneously improve the CAT-like activity of Pd@Au-PEG NSs and initiate moderate PTT for the long-term modulation of hypoxic TME (Additional file 1: Fig. S15). Illustrated through the average tumor growth curves and end-of-treatment tumor photographs for each treatment group, it was evident that while Pd@Au-PEG NSs and moderate PTT in isolation did not exert a significant inhibitory effect on tumor growth, they effectively played as synergistic cofactors of RIT to enhance its tumor-ablating efficacy (Fig. 4b; Additional file 1: Figure S16a). Despite ^90^Y resin spheres at the radioactivity dose of 3.7 MBq could suppress tumor progression, the absence of a substantial cure rate (0%) pointed towards the inadequacy of the employed radioactivity dosage in achieving complete tumor eradication, which may be associated with the hypoxia-resulted insufficient DNA disruption [21]. In contrast, with the crucial aid of hypoxia relief and moderate PTT, RIT with the same radioactivity dosage of ^90^Y resin spheres achieved noteworthy ablation of 4T1 subcutaneous tumors, attaining a cure rate of 80% (Additional file 1: Fig. S17). Furthermore, the analysis of tumor sample weights obtained from each group at the conclusion of treatment also confirmed the remarkable performance of this enhanced RIT strategy (Additional file 1: Fig. S16b).

Our investigations also revealed that both singular RIT and RIT augmented by hypoxic TME modulation exhibited a remarkable improvement in the survival rate of tumor-bearing mice. This enhancement was attributed to the tumor-suppressing effect induced by the high-energy β-radiation of ^90^Y (β_average_ = 933.7 keV) [50], as depicted in Fig. 4c. Notably, mice with tumors exceeding 1000 mm^3^ were recorded as deceased in our experimental protocol, and the termination point of the therapeutic experiment was determined by the point at which all tumor-bearing mice in any group died. Although the observed survival rates for both group III and group IV reached 100% over the 16-day observation period, it should be highlighted that the tumor size of mice in group III would undoubtedly and more rapidly surpass 1000 mm^3^ if the observation duration were extended due to the suboptimal tumor ablation efficacy inherent in the single RIT treatment at the provided dosage. Simultaneously, the weights of mice in all four treatment groups were also monitored alongside the measurement of each tumor’s size. This comprehensive assessment was conducted to evaluate the biosafety associated with each therapeutic modality. Comparative analysis against the control group demonstrated that the weight variations of mice subjected to interventions involving Pd@Au-PEG NSs + laser, intratumoral implantation of ^90^Y resin spheres, or their combined application adhered to the expected normal trends (Fig. 4d).

The results of hematoxylin-eosin (H&E) staining of residual tumor samples from group IV mice clearly exhibited noticeably incomplete tissue morphology. This stood in stark contrast to the staining results of group III, which depicted the limited cancer cell-destructive efficacy of single RIT (Fig. 4e (top panel)). To delve further into the substantial cell necrosis induced by hypoxia relief-boosted RIT, we proceeded with the assay employing TdT-mediated dUTP nick-end labeling (TUNEL) and Ki67 staining. In the parallel comparison of TUNEL staining images, the most intense green fluorescence indicative of pronounced cell apoptosis was conspicuously present in group IV (Fig. 4e (middle panel); Additional file 1: Fig. S18). Additionally, we conducted the examination of Ki67, a cell cyclin-related protein that serves as a marker for profiling cell apoptosis and proliferation, to reveal the proliferating activity of cancer cell post various treatment. Generally, Ki67 provides a reliable reflection of the proliferative activity of tumor cells, and its expression is closely linked to the development, metastasis, and prognosis of various types of tumors [51, 52]. As depicted, Group IV displayed the least expression of Ki67, further demonstrating the superior proliferation-inhibiting effect of this RIT-boosting approach (Fig. 4e (bottom panel)). In conclusion, we successfully pioneered a simple yet highly effective method to amplify RIT efficacy. This innovative strategy relies on the efficiently long-term TME hypoxia modulation achieved by biocompatible CAT-like Pd@Au-PEG NSs coupled with NIR-II moderate PTT. It presents an appealing alternative to remove the hypoxia-induced limitations on the therapeutic outcome of clinical radioactive agents.

### The histological evaluation of antitumor effect and biosafety of combined therapy

To histologically exhibit the safety profile and tumor necrosis-inducing efficacy associated with each treatment modality, we initiated a comprehensive pathological investigation encompassing major organs such as heart, liver, spleen, lung, and kidney. This was accomplished through H&E staining of tissue samples obtained from mice within each treatment group. Upon comparative analysis with the control group, it became evident that interventions involving intratumoral injection of ^90^Y resin spheres, systemic administration of Pd@Au-PEG NSs, or their combined application did not give rise to pronounced tissue damage in the healthy organs (Additional file 1: Fig. S19). This clarified the excellent biocompatibility of our TME-modulating strategy and the synergistic therapeutic method. Meanwhile, the detection of complete blood count parameters of Pd@Au-PEG NSs-injected mice also showed minimal deviations compared to the saline-injected mice, thereby confirming the good biocompatibility of Pd@Au-PEG NSs (Additional file 1: Fig. S20).

## Conclusions

Recognizing the mismatch between brief hypoxia relief and the long-acting RIT, we introduce an approach that ensures continuous and efficient hypoxia alleviation to boost the tumor-ablating efficiency of RIT. Elaborately engineered 2D Pd@Au-PEG NSs, featuring robust CAT-like activity and NIR-II photothermal conversion capability, can persistently decompose H_2_O_2_ into O_2_, consequently mitigating hypoxia within solid tumors. Through the tumor-localized NIR-II irradiation, both the intratumoral Pd@Au-PEG NSs’ enzyme-mimicking activity and tumor blood perfusion experience enhancement. This two-pronged tactic works synergistically to optimize the efficacy of TME hypoxia modulation, significantly enhancing the tumor ablation outcomes of ^90^Y resin spheres-based RIT. This study aims to draw attention to the critical importance of achieving prolonged hypoxia relief to potentiate the tumoricidal effect of clinical RIT agents, including the tumor-targeting radiopharmaceuticals and biocompatible interventional treatment devices with radiolabeling.

## Experimental section

### Materials

Pd(acac)_2_, Au(PPh_3_)Cl, N,N-dimethylacetamide, N,N-dimethylformamide (DMF), polyvinyl pyrrolidone (PVP), NaBr were purchased from the Sigma-Aldrich (Shanghai, China). Cy5.5-NHS and Hoechst were purchased from Shanghai Aladdin Biochemical Technology Co., Ltd. SH-mPEG and NH_2_-PEG-SH were obtained from Shanghai Ziqi Biotechnology Co., Ltd. Calcein-acetoxymethyl ester (calcein-AM) and propidium iodide (PI) assay kit were obtained from Yeasen Biotechnology (Shanghai) Co., Ltd. All chemicals and reagents used in this work were analytical grade and used without any further purification. The radiopharmaceutical ^90^Y resin microspheres were provided by the First Affiliated Hospital of School of Medicine of Zhejiang University.

### Synthesis and PEGylation of Pd@Au NSs

The preparation of Pd@Au-PEG NSs followed the two-steps seed growing method in our previous work with a slight modification. Firstly, for the synthesis of original seed (13 nm Pd NSs), Pd(acac)_2_ (10 mg), PVP (32 mg) and NaBr (10 mg) were mixed with N,N-dimethylacetamide (2 mL) and deionized (DI) water (4 mL) in a glass pressure vessel to obtain a homogeneous reaction mixture. Then, the vessel was charged with CO (1 bar) and heated from room temperature to 60 °C within 30 min, and then kept at 60 °C for 150 min. Next, Pd(acac)_2_ (37.5 mg) was added to 13 nm Pd NSs seeds solution. Under the condition of CO (1 bar), the reaction mixture was heated from room temperature to 60 °C within 60 min, and then kept at 60 °C for 60 min to fabricate the second-step seeds (30 nm Pd NSs). Finally, 30 nm Pd NSs were washed by acetone-ethanol and dispersed in DI water (2 mL), then the solution was mixed with Au(PPh_3_)Cl (4 mL, 0.75 mg mL^− 1^ in DMF). The hydrazine was chosen as reductant and added dropwise into the above mixture under stirring. After standing at room temperature for 12 h, the final products were collected by centrifugation and redispersed in saline or DI water for further use. Owing to the strong binding between Pd/Au and sulfhydryl group, SH-mPEG (Molecular weight = 5000 kDa, 20 mg) was added into the above-mentioned solution of Pd@Au NSs for their surface PEGylation.

### Materials characterizations

The UV–Vis–NIR absorption spectra of Pd@Au-PEG NSs were recorded using the Cary 5000 Scan UV-Vis-NIR spectrophotometer (Varian Medical Systems, Palo Alto, CA, USA). All the transmission electron microscopy (TEM) images were acquired using the JEM-2100 TEM operating at 300 kV. The hydrodynamic size and surface Zeta potential of Pd@Au-PEG NSs were obtained from Malvern Zeta-sizer nano ZS (Malvern Instruments Ltd) for parallel three times.

### The measurement of catalase activity and stability of Pd@Au-PEG NSs

The catalase-like activity of prepared Pd@Au-PEG NSs was evaluated by real-time monitoring the content of dissolved O_2_ in the hydrogen peroxide (H_2_O_2_) aqueous solution (15 mL, 30 mM) incubated with Pd@Au-PEG NSs (250 ppm) using dissolved O_2_ meter (JPBJ-609 L, Shanghai INESA scientific instrument Co., Ltd). To clarify the continuously O_2_ producing ability of Pd@Au-PEG NSs, we repeatedly added H_2_O_2_ (5 mM) into the aqueous solution when the H_2_O_2_ had been decomposed completely followed by the persistent measurement of dissolved O_2_ content.

To investigate the boosting effect of NIR-II laser irradiation on the catalase-like activity of Pd@Au-PEG NSs, the aqueous solution containing H_2_O_2_ and Pd@Au-PEG NSs with the same concentration as above was irradiated by 1064 nm laser (0.3 W cm^− 2^), and the sample without lase irradiation was set as control group. The variation of dissolved O_2_ contents with time in these two samples were monitored in turn. Furthermore, the Pd@Au-PEG NSs’ activity under the different pH conditions (pH = 7, 4.5 or 3.5) to was studied to demonstrate their robust tolerance to harsh TME. Taking the neutral (pH = 7) condition as the control group (100%), the Pd@Au-PEG NSs’ activity under other two pH conditions were calculated and compared simultaneously.

### The evaluation of NIR-II photothermal conversion of Pd@Au-PEG NSs

Encouraged by the NIR-II optical window of Pd@Au-PEG NSs, we assessed the NIR-II photothermal converting ability. Briefly, the aqueous solutions of Pd@Au-PEG NSs with different concentration (10, 20, 40 and 80 ppm) were irradiated by 1064 nm laser (0.3 W cm^− 2^), and the increasing temperature values and images with a 0.5 min interval were recorded using hand-in infrared thermal imaging instrument. The experiment regarding the NIR-II photothermal conversion of Pd@Au-PEG NSs (40 ppm) under the conditions of various laser power density (0.1, 0.3, 0.5 and 0.7 W cm^− 2^) was operated similarly as the protocol above. The NIR-II photothermal stability of Pd@Au-PEG NSs was tested by the multi-times repeated “laser heating-cooling” cycle. Specifically, the Pd@Au-PEG NSs solution (40 ppm) was irradiated by the NIR-II laser (1064 nm, 0.3 W cm^− 2^) to perform the first time photothermal conversion. When the solution temperature reached around 45 ℃, the laser was turned off to make the solution temperature decrease to below 30 ℃. Then, the laser was turned on again to repeat the photothermal conversion experiment of the same Pd@Au-PEG NSs sample. This cycling operation was conducted for six times, and the increasing and decreasing temperature values were recorded with a 0.25 min interval.

### Cytotoxicity assay

The cell biocompatibility of Pd@Au-PEG NSs and the in vitro therapeutic effect were examined via standard CCK8 experiment using 4T1 cancer cells. 4T1 cells were cultured at 37 °C in the humidified air atmosphere with 5% CO_2_ in RPMI 1640 medium, which contained 10% fetal bovine serum (FBS), penicillin (100 U mL^− 1^) and streptomycin (100 μg mL^− 1^). After adding Pd@Au-PEG NSs solution with increasing concentrations and co-cultured for 24 h under the uniform condition as above, 100 μL medium containing 5 μg CCK8 reagent was added to each well. Then, the cell-culturing plate was incubated for 1 h at 37 °C and subsequently being shaken at room temperature for 10 min. Finally, the cytotoxicity of the Pd@Au-PEG NSs was analyzed by detecting the absorbance of each well at 450 nm. In vitro therapeutic effect was also evaluated by substituting the co-cultured samples with planned agents and treatments (Pd@Au-PEG (30 μg mL^− 1^), ^90^Y (1.48 MBq mL^– 1^) and NIR-II laser (0.3 W cm^− 2^)) and using the CCK8-based cell-viability detecting approach and Calcein-AM (green)/PI (red) dual-staining fluorescence microscopy. All cell groups were cultured under the hypoxic condition.

### Western blotting analysis

Western blotting was conducted to quantify the protein expression of hypoxia-inducible factor-1α (HIF-1α, Abcam, AB199) in 4T1 cells. Initially, 4T1 cells were seeded in 6-well plates and incubate overnight. The cells were then divided into three groups: control, Pd@Au-PEG NSs, and Pd@Au-PEG NSs + laser (1064 nm, 0.3 W cm^− 2^, with the maximum temperature maintained at 45 °C). Subsequently, the cells were treated with H_2_O_2_ (100 μM) for a duration of 24 h. To induce hypoxia, the 4T1 cells under different treatments were placed in a hypoxic incubator (Thermo Scientific) maintained at 1.8% O_2_ and 5% CO_2_ (with the remaining balance being N_2_) for a period of 24 h. Cells were harvested and lysed in RIPA buffer containing 1% phosphatase and protease inhibitors, followed by boiling for 10 min at 100 °C. The proteins were subsequently separated using SDS-PAGE electrophoresis and transferred onto Immobilon PVDF membranes (Millipore). The membrane was then blocked with 5% milk in TBST for one hour. The primary antisera against HIF-1α were diluted by 1000 and incubated overnight at 4 °C in the blocking buffer. Subsequently, the membranes were incubated at room temperature for two hours with the secondary antibody. Protein signals were detected using an ECL system (Amersham Biosciences, Buckinghamshire, UK). β-actin were used as internal controls.

### The construction of 4T1 subcutaneous tumor models

Balb/c mice (female, 5 weeks, 18 ~ 20 g body weight) and Balb/c nude mice (female, 5 weeks, 18 ~ 20 g body weight, for fluorescence imaging) were obtained from the animal center of the First Affiliated Hospital of School of Medicine of Zhejiang University. The 4T1 subcutaneous tumor models were performed by subcutaneous injection (~ 1 × 10^6^ HepG2 cells in 75 μL cell culture medium without serum) on the right rear legs of experimental mice. After inoculation for around 1 week, the tumor volume will reach about 80 mm^3^ ~ 100 mm^3^, which could be used for the living imaging and in vivo treatment research.

### In vivo multi-modal imaging

The photoacoustic (PA) imaging and computed tomography (CT) imaging of Pd@Au-PEG NSs-injected mice (n = 3) were performed on the Vevo LAZR-X Imaging System (FUJIFILM VisualSonics Inc.) at the excitation wavelength of 970 nm and PET-CT imaging instrument (Inveon scanner (Siemens, USA)), respectively. Specifically, Pd@Au-PEG NSs solution (250 μL, 1 mg mL^− 1^) was intravenous administrated on 4T1 tumor-bearing mice followed by PA and CT signals acquisition at various time points post-injection.

The fluorescence imaging was operated on the live animal optical imaging system (IVIS Lumina II). Firstly, Cy5.5-NHS was covalently linked to the Pd@Au NSs with NH_2_-PEG-SH modification in PBS (pH = 8) to afford Pd@Au-Cy5.5 NSs as the fluorescence probes, which were purified via ultrafiltration to remove the excessive Cy5.5-NHS. Then, Pd@Au-Cy5.5 NSs were injected intravenously on 4T1 tumor-bearing nude mice (n = 3), and the whole-body living fluorescence imaging signals were acquired at 6 and 24 h post-injection.

### In vivo therapeutic effect evaluation

4T1 tumor-bearing mice were randomly divided into four groups (n = 5), namely, saline group (Group I), Pd@Au-PEG NSs + laser group (Group II), ^90^Y resin spheres group (Group III) and ^90^Y resin spheres + Pd@Au-PEG NSs + laser (Group IV). Taking the group IV as the example, Pd@Au-PEG NSs solution (250 μL, 1 mg mL^− 1^) was injected intravenously. Then, ^90^Y resin spheres (3.7 MBq) was intratumorally implanted to execute RIT at 24 h post-injection of Pd@Au-PEG NSs. Subsequently, multiple NIR-II moderate PTT under the laser (1064 nm, 0.3 W cm^− 2^) excitation was operated at day -1, -3 and -5 post-injection of Pd@Au-PEG NSs by irradiating the tumor for about 4 min to make the temperature of tumor areas reach ~ 45 °C, thus achieving the augment of catalase-like activity and the improvement of tumor blood perfusion. The experimental protocols of group II and group III were similar to those of group IV, without the procedure of ^90^Y resin spheres implantation, or Pd@Au-PEG NSs + moderate PTT, respectively. The tumor sizes and weights of all mice were recorded per 2 days.

### Statistical analysis

All the data were presented as the mean (±) standard deviation (SD). The statistical analyses were operated and plotted using OriginPro 2023b software. SD is indicated by the error bars. Asterisks stand for significant differences (*P < 0.05, **P < 0.01 and ***P < 0.001).

### Electronic supplementary material

Below is the link to the electronic supplementary material.

**Additonal file 1: Figure S1:** TEM image of 30 nm Pd NSs. **Figure S2:** Quantitative content analysis of Au and Pd in Pd@Au NSs by ICP-MS analysis. **Figure S3:** UV-Vis-NIR absorption spectrum of (a) Pd-PEG NSs and (b) Pd@Au-PEG NSs. **Figure S4:** Zeta potentials comparison of Pd@Au NSs and Pd@Au-PEG NSs. **Figure S5:** Hydrodynamic sizes of (a) Pd@Au NSs and (b) Pd@Au-PEG NSs incubated in the saline at day-1, -3, -5 and -7. **Figure S6:** The absorption spectrum of Pd@Au-PEG NSs incubated in the saline at day-1, -3, -5 and  -7. **Figure S7:** Enzymatic activity of Pd@Au-PEG NSs under various pH conditions. **Figure S8:** (a) Photothermal converting ability of Pd@Au-PEG NSs with various concentrations under the excitation of NIR-II laser (1064 nm, 0.3 W cm^− 2^). (b) NIR-II photothermal converting ability of Pd@Au-PEG NSs (40 ppm) under the excitation of NIR-II laser with various power density. **Figure S9:** TEM image of Pd@Au-PEG NSs after photothermal stability test (Scale bar = 50 nm). **Figure S10:** The subcellular localization of Cy5.5-labeled Pd NSs (Scale bar = 10 µm). **Figure S11:** In vitro PA images of Pd@Au-PEG NSs with various concentrations (0, 16, 32, 63, 125, 250 and 500 ppm) and the linear relationship between the PA signal intensity and Pd@Au-PEG NSs concentration. **Fig. S12:** In vitro CT images of Pd@Au-PEG NSs with various concentrations (0, 20, 40, 80, 100, 160 and 200 ppm) and the linear relationship between the CT signal intensity and Pd@Au-PEG NSs concentration. **Fig. S13:** Small animal living fluorescence imaging of Pd@Au-Cy5.5 NSs-injected mice at 6 h and 24 h post-injection (Red circles and arrows represent the tumor sites). **Fig. S14:** (a) In vitro fluorescence images of major organs obtained from Pd@Au-Cy5.5-injected mice at 24 h post-injection (H: Heart, Li: Liver, S: Spleen; Lu: Lung, K: Kidney, T: Tumor). (b) The quantitative analysis of tissue fluorescence intensity. **Fig. S15:***In vivo* NIR-II moderate PTT images of Pd@Au-PEG NSs-injected mice. **Fig. S16:** (a) The size of tumors at 16th days post-treatment. (b) The weight of tumor samples at 16th days post-treatment from each treatment groups. **Fig. S17:** The average growth curves of 4T1 subcutaneous tumors treated by different therapeutic modalities. **Fig. S18:** The quantitative analysis of TUNEL fluorescence intensities in each treatment group. **Fig. S19:** H&E staining images of major health tissues including heart, liver, spleen, lung and kidney of mice in each treatment group (Scale bar = 50 µm). **Fig. S20:** Hematological index measurements of Pd@Au-PEG NSs-injected mice

## Data Availability

No datasets were generated or analysed during the current study.

## Data Availability

The data and materials used in this study are available from the corresponding author upon reasonable requests.
