# Supplementary material for: A continuously efficient O2-supplying strategy for long-term modulation of hypoxic tumor microenvironment to enhance long-acting radionuclides internal therapy
Source: J Nanobiotechnology. 2024 Jan 3;22:7. doi: 10.1186/s12951-023-02268-5 (PMC10763042; doi:10.1186/s12951-023-02268-5)
Supplement: Supplementary file 1 — Additonal file 1: Figure S1: TEM image of 30 nm Pd NSs. Figure S2: Quantitative content analysis of Au and Pd in Pd@Au NSs by ICP-MS analysis. Figure S3: UV-Vis-NIR absorption spectrum of (a) Pd-PEG NSs and (b) Pd@Au-PEG NSs. Figure S4: Zeta potentials comparison of Pd@Au NSs and Pd@Au-PEG NSs. Figure S5: Hydrodynamic sizes of (a) Pd@Au NSs and (b) Pd@Au-PEG NSs incubated in the saline at day-1, -3, -5 and -7. Figure S6: The absorption spectrum of Pd@Au-PEG NSs incubated in the saline at day-1, -3, -5 and -7. Figure S7: Enzymatic activity of Pd@Au-PEG NSs under various pH conditions. Figure S8: (a) Photothermal converting ability of Pd@Au-PEG NSs with various concentrations under the excitation of NIR-II laser (1064 nm, 0.3 W cm− 2). (b) NIR-II photothermal converting ability of Pd@Au-PEG NSs (40 ppm) under the excitation of NIR-II laser with various power density. Figure S9: TEM image of Pd@Au-PEG NSs after photothermal stability test (Scale bar = 50 nm). Figure S10: The subcellular localization of Cy5.5-labeled Pd NSs (Scale bar = 10 µm). Figure S11: In vitro PA images of Pd@Au-PEG NSs with various concentrations (0, 16, 32, 63, 125, 250 and 500 ppm) and the linear relationship between the PA signal intensity and Pd@Au-PEG NSs concentration. Fig. S12: In vitro CT images of Pd@Au-PEG NSs with various concentrations (0, 20, 40, 80, 100, 160 and 200 ppm) and the linear relationship between the CT signal intensity and Pd@Au-PEG NSs concentration. Fig. S13: Small animal living fluorescence imaging of Pd@Au-Cy5.5 NSs-injected mice at 6 h and 24 h post-injection (Red circles and arrows represent the tumor sites). Fig. S14: (a) In vitro fluorescence images of major organs obtained from Pd@Au-Cy5.5-injected mice at 24 h post-injection (H: Heart, Li: Liver, S: Spleen; Lu: Lung, K: Kidney, T: Tumor). (b) The quantitative analysis of tissue fluorescence intensity. Fig. S15:In vivo NIR-II moderate PTT images of Pd@Au-PEG NSs-injected mice. Fig. S16: (a) The size of tumors at 1 [file 12951_2023_2268_MOESM1_ESM.docx]

Supporting Information

**A Continuously Efficient O_2_-Supplying Strategy for Long-Term Modulation of Hypoxic Tumor Microenvironment to Enhance Long-Acting Radionuclides Internal Therapy**

Jingchao Li^1#^, Tingting Wang^1#^, Yuanfei Shi^3#^, Zichen Ye^2^, Xun Zhang^1^, Jiang Ming^2^, Yafei Zhang^1^, Xinyan Hu^2^, Yun Li^4^, Dongsheng Zhang^1^, Qianhe Xu^1^, Jun Yang^1^, Xiaolan Chen^2*^, Nian Liu^1*^, Xinhui Su^1*^

^1^ Department of Nuclear Medicine, The First Affiliated Hospital, Zhejiang University School of Medicine, Hangzhou 310003, China.

^2^ State Key Laboratory for Physical Chemistry of Solid Surfaces, Collaborative Innovation Center of Chemistry for Energy Materials, and Engineering Research Center for Nano-Preparation Technology of Fujian Province, College of Chemistry and Chemical Engineering, Xiamen University, Xiamen 361005, China.

^3^ Department of Hematology, The First Affiliated Hospital, Zhejiang University School of Medicine, Hangzhou 310003, China.

^4^ State Key Laboratory of Molecular Vaccinology and Molecular Diagnostics & Center for Molecular Imaging and Translational Medicine, School of Public Health, Xiamen University, Xiamen 361005, China.

^*^Correspondence: chenxl@xmu.edu.cn, nian.liu@zju.edu.cn, suxinhui@zju.edu.cn


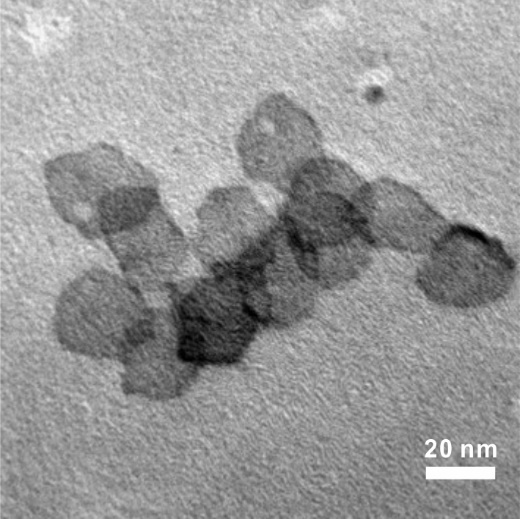


**Figure S1**: TEM image of 30 nm Pd NSs.


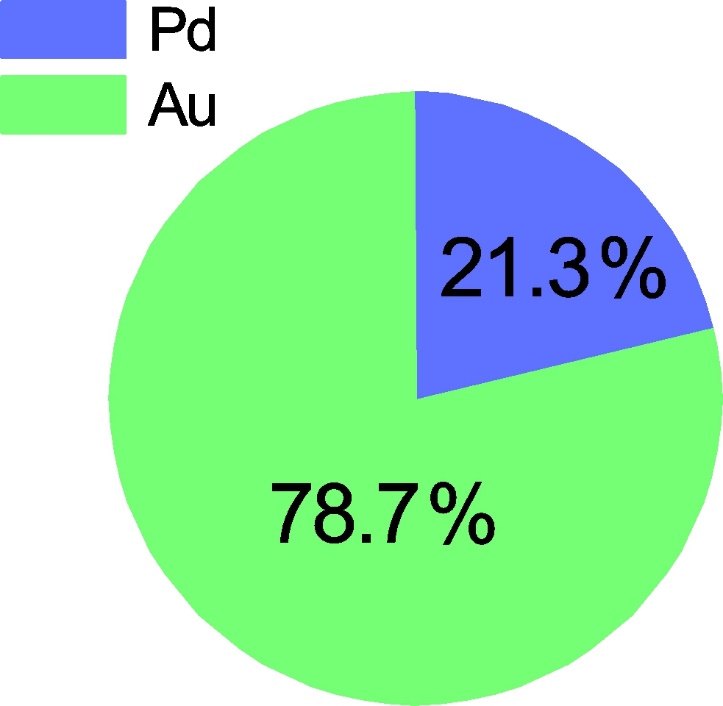


**Figure S2**: Quantitative content analysis of Au and Pd in Pd@Au NSs by ICP-MS analysis.


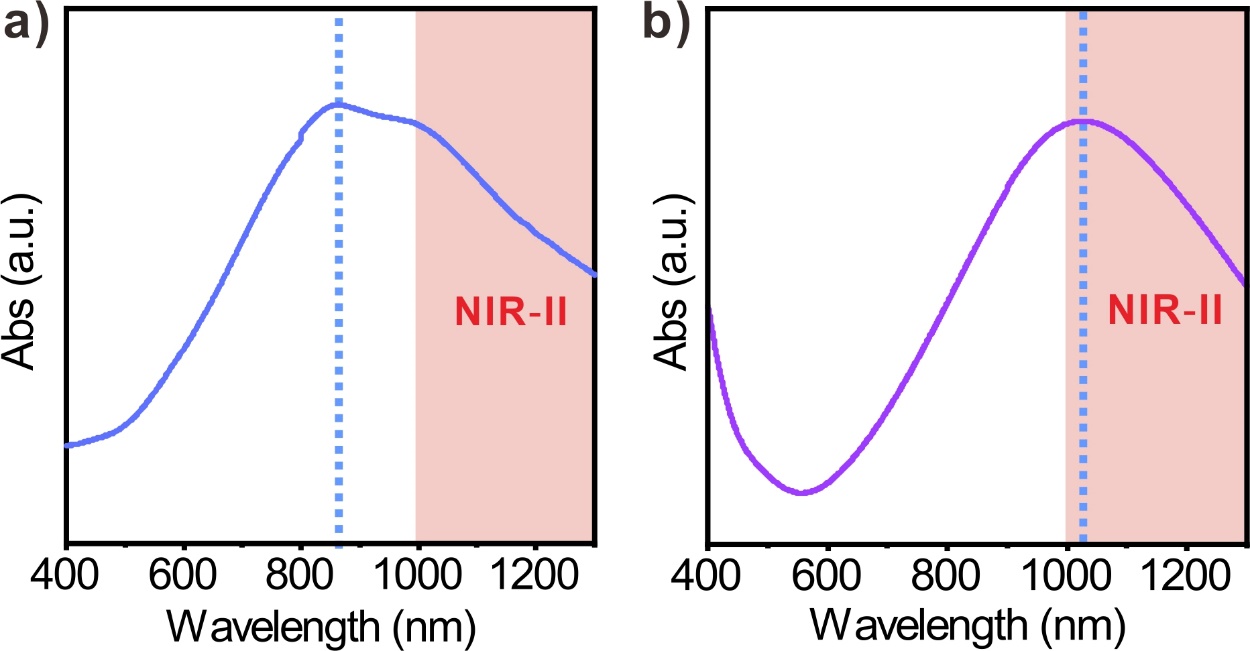


**Figure S3**: UV-Vis-NIR absorption spectrum of (a) Pd-PEG NSs and (b) Pd@Au-PEG NSs.


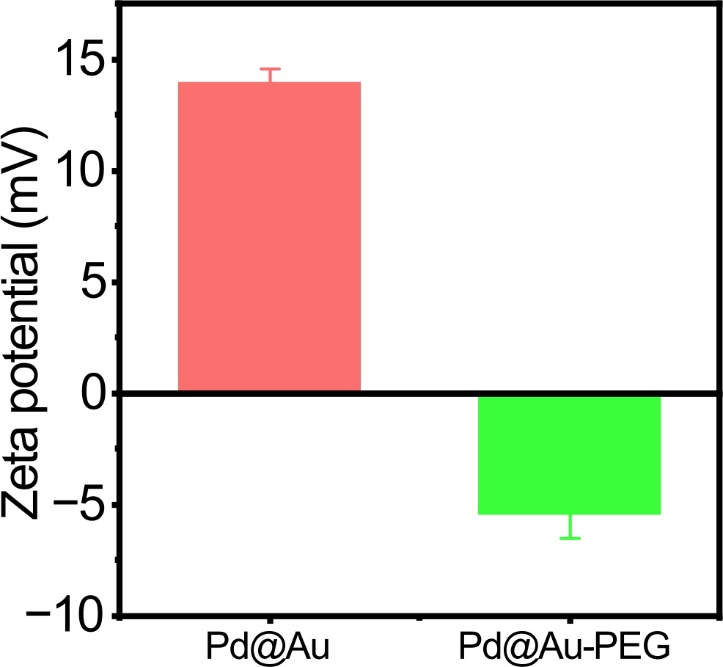


**Figure S4**: Zeta potentials comparison of Pd@Au NSs and Pd@Au-PEG NSs.


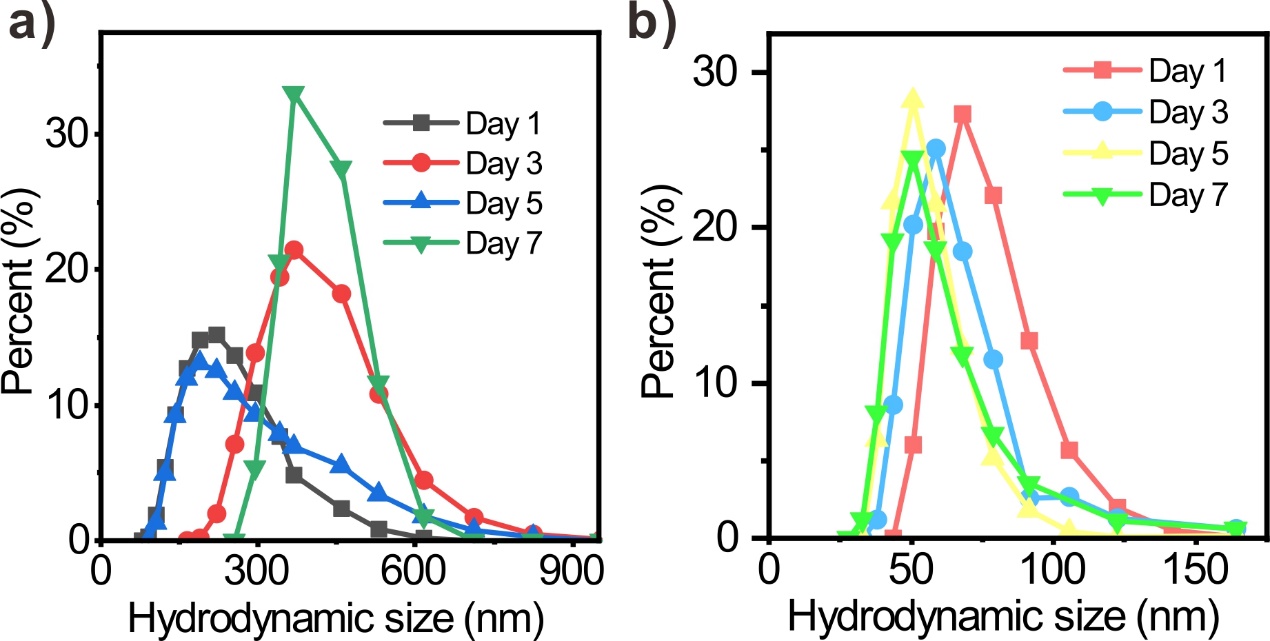


**Figure S5**: Hydrodynamic sizes of (a) Pd@Au NSs and (b) Pd@Au-PEG NSs incubated in the saline at day-1, -3, -5 and -7.


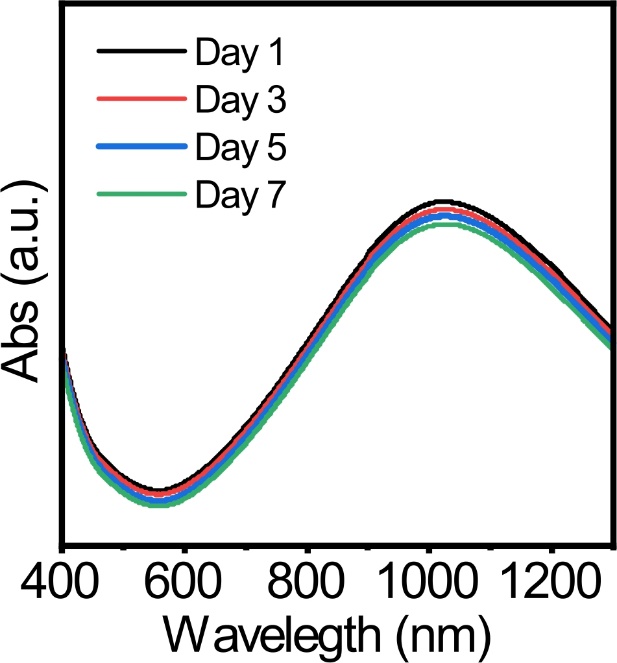


**Figure S6**: The absorption spectrum of Pd@Au-PEG NSs incubated in the saline at day-1, -3, -5 and -7.


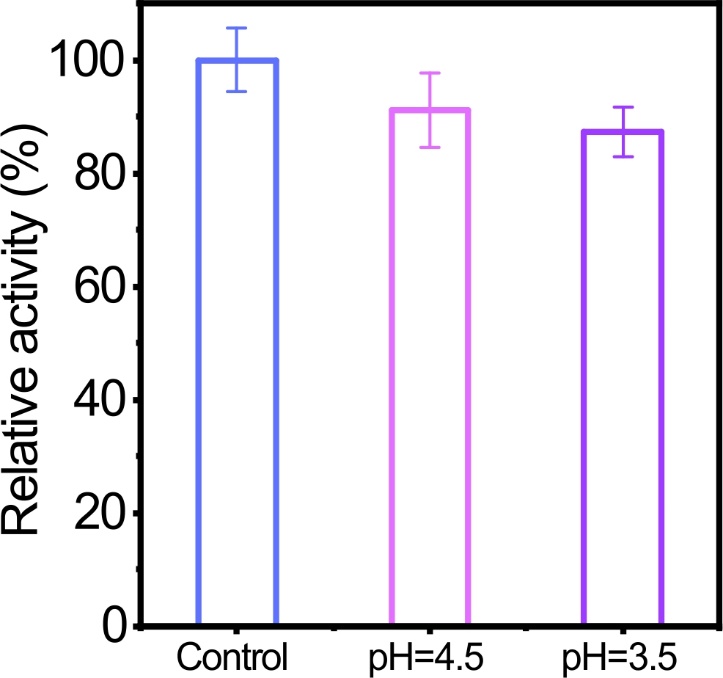


**Figure S7**: Enzymatic activity of Pd@Au-PEG NSs under various pH conditions.


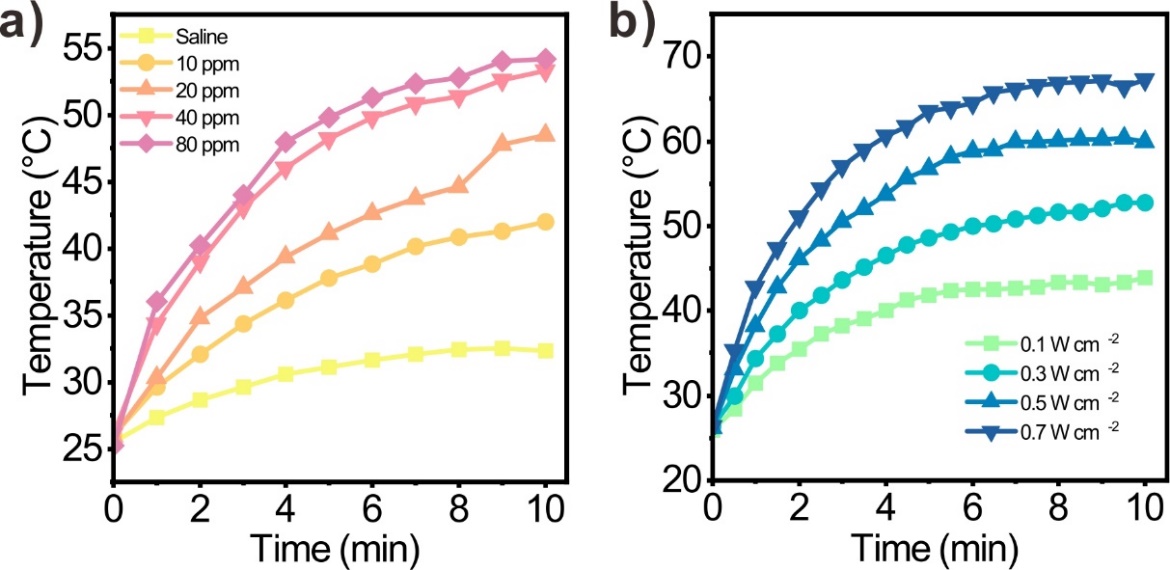


**Figure S8**: (a) Photothermal converting ability of Pd@Au-PEG NSs with various concentrations under the excitation of NIR-II laser (1064 nm, 0.3 W cm^-2^). (b) NIR-II photothermal converting ability of Pd@Au-PEG NSs (40 ppm) under the excitation of NIR-II laser with various power density.


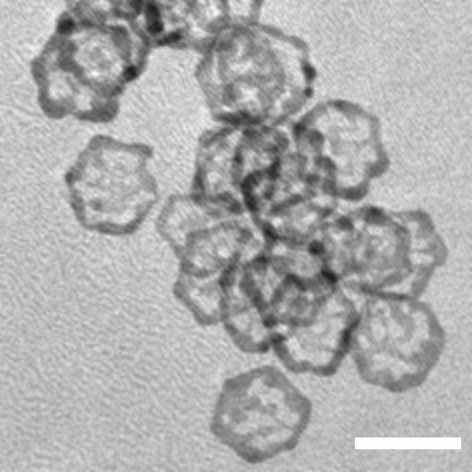


**Figure S9**: TEM image of Pd@Au-PEG NSs after photothermal stability test (Scale bar = 50 nm).


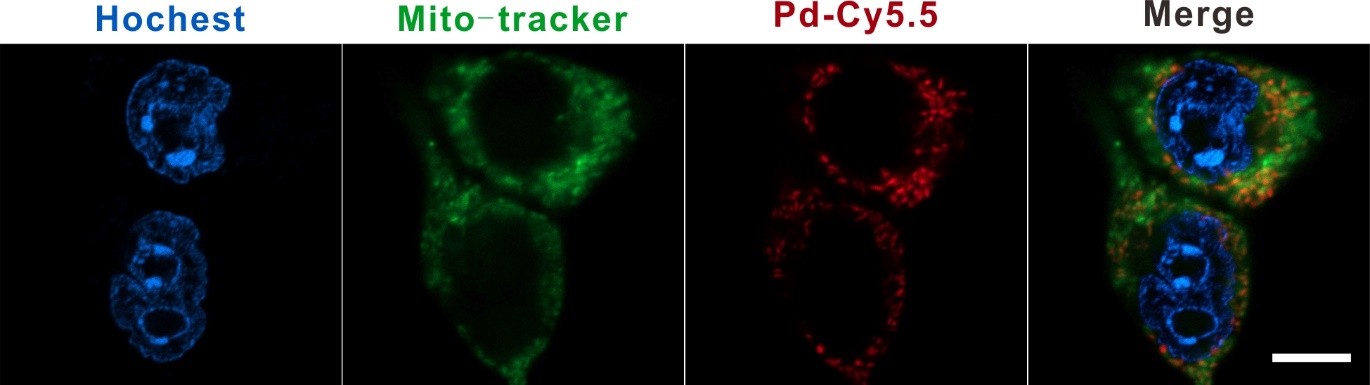


**Figure S10**: The subcellular localization of Cy5.5-labeled Pd NSs (Scale bar = 10 μm).


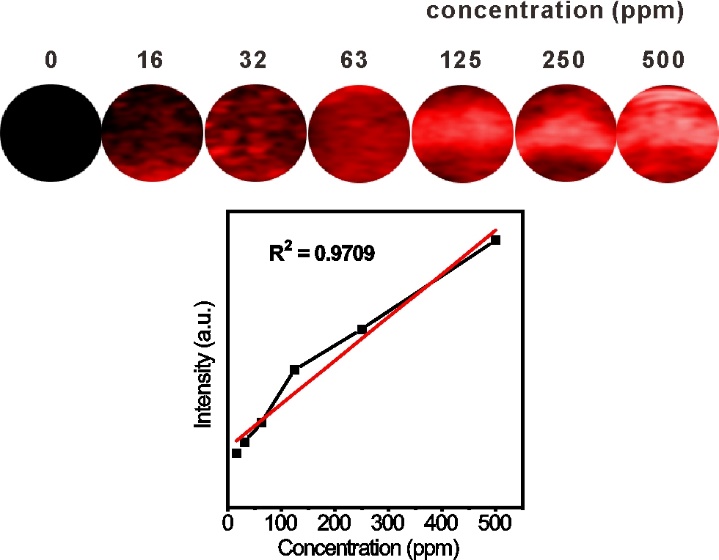


**Figure S11**: *In vitro* PA images of Pd@Au-PEG NSs with various concentrations (0, 16, 32, 63, 125, 250 and 500 ppm) and the linear relationship between the PA signal intensity and Pd@Au-PEG NSs concentration.


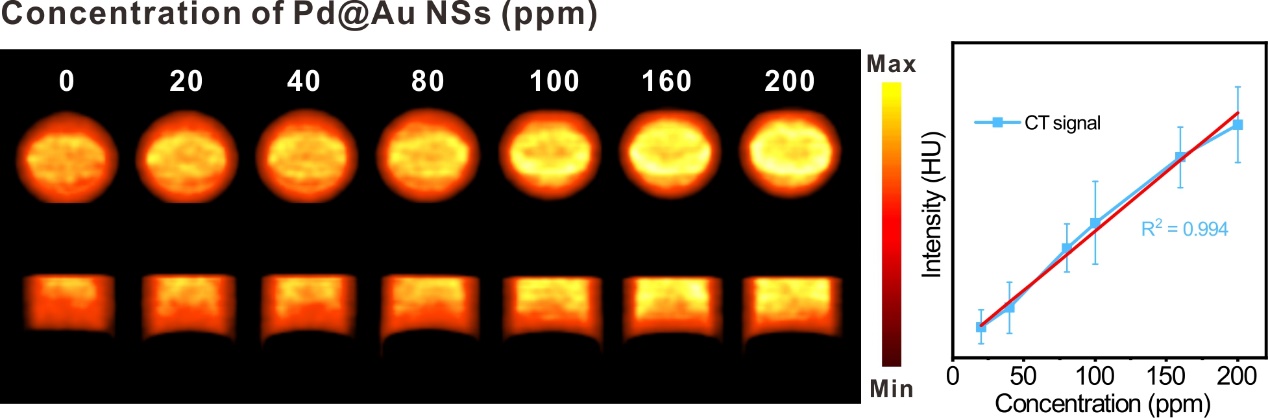


**Fig. S12**: *In vitro* CT images of Pd@Au-PEG NSs with various concentrations (0, 20, 40, 80, 100, 160 and 200 ppm) and the linear relationship between the CT signal intensity and Pd@Au-PEG NSs concentration.


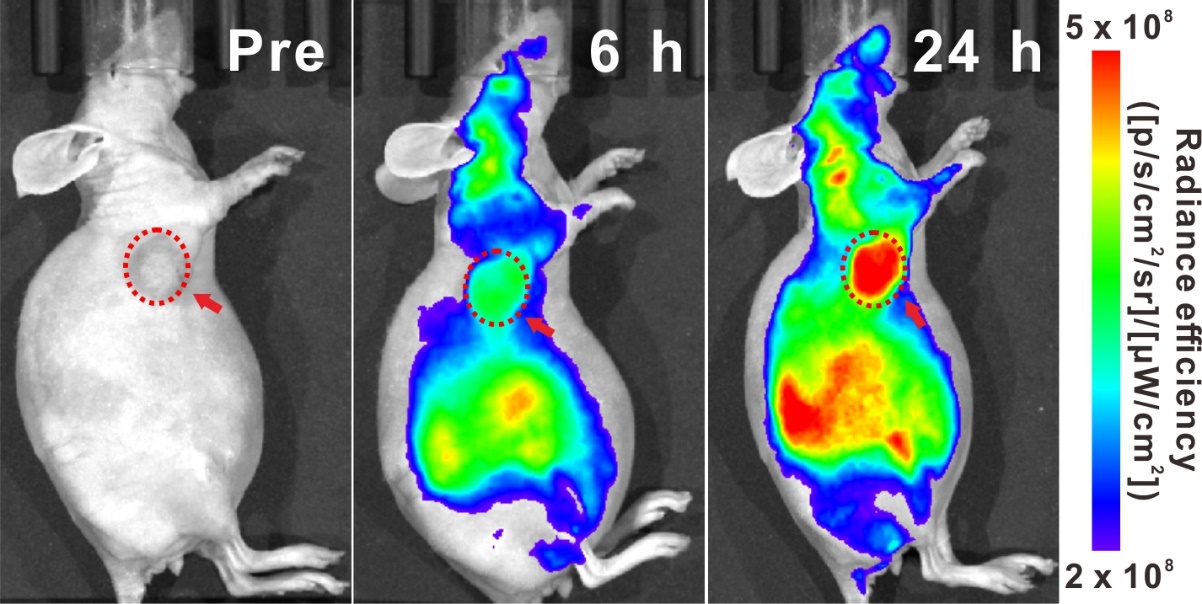


**Fig. S13**: Small animal living fluorescence imaging of Pd@Au-Cy5.5 NSs-injected mice at 6 h and 24 h post-injection (Red circles and arrows represent the tumor sites).


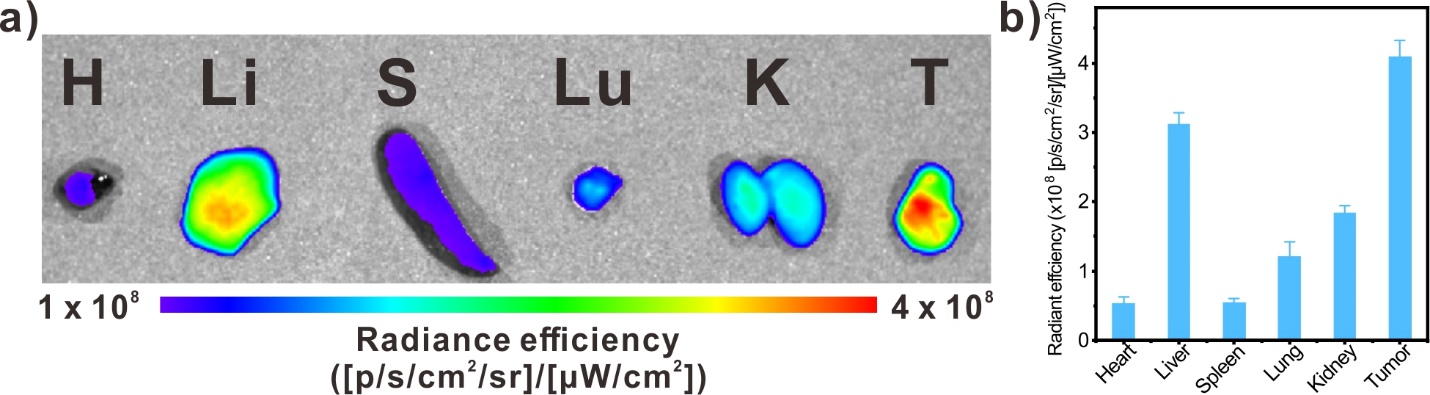


**Fig. S14**: (a) *In vitro* fluorescence images of major organs obtained from Pd@Au-Cy5.5-injected mice at 24 h post-injection (H: Heart, Li: Liver, S: Spleen; Lu: Lung, K: Kidney, T: Tumor). (b) The quantitative analysis of tissue fluorescence intensity.


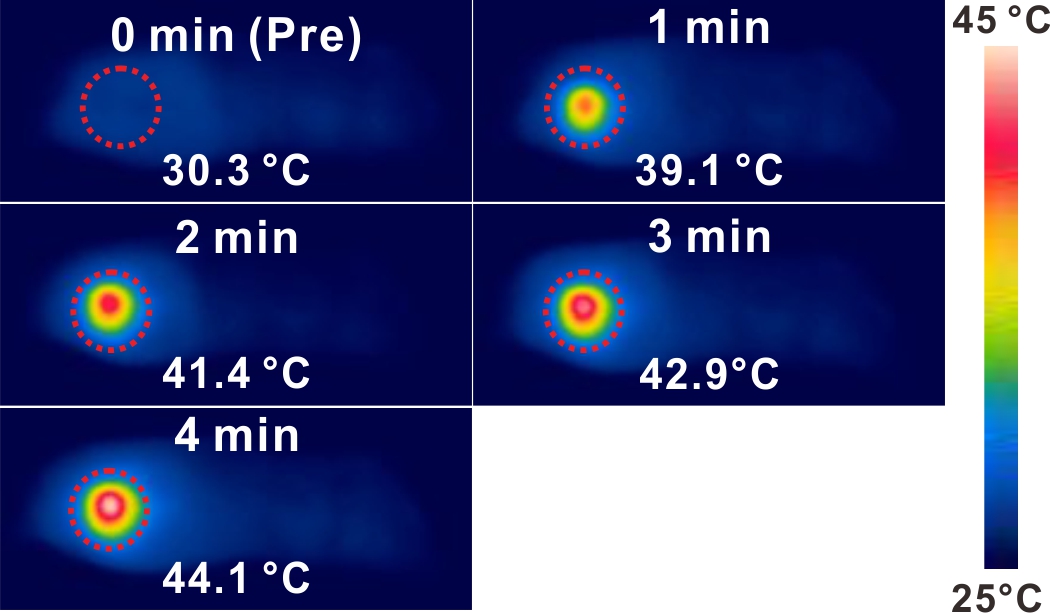


**Fig. S15**: *In vivo* NIR-II moderate PTT images of Pd@Au-PEG NSs-injected mice.


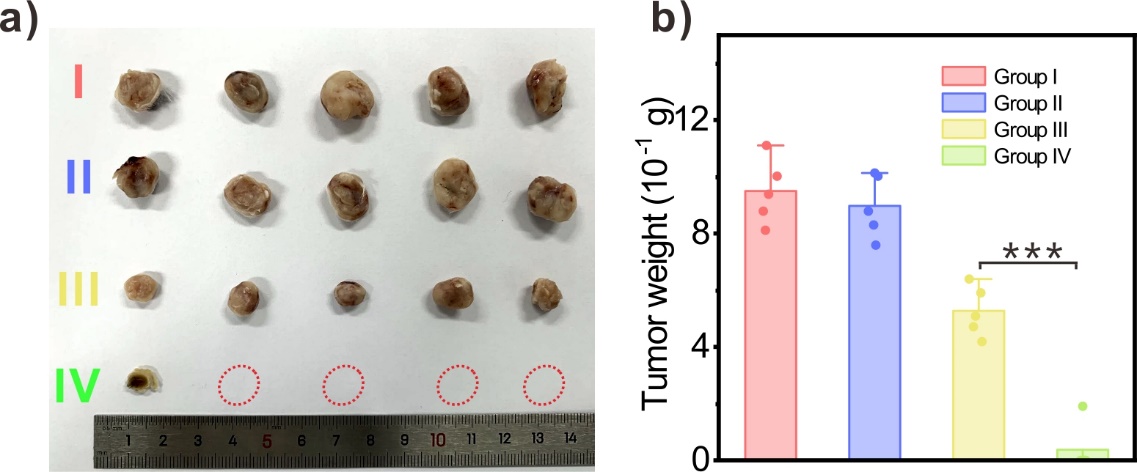


**Fig. S16**: (a) The size of tumors at 16^th^ days post-treatment. (b) The weight of tumor samples at 16^th^ days post-treatment from each treatment groups.


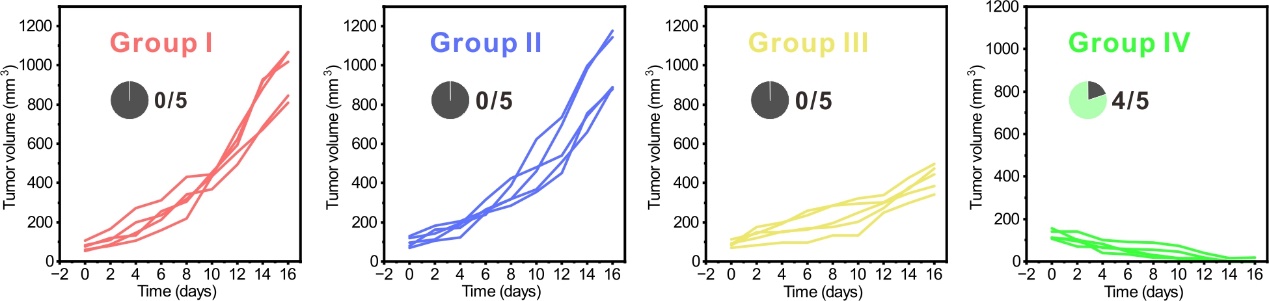


**Fig. S17**: The average growth curves of 4T1 subcutaneous tumors treated by different therapeutic modalities.


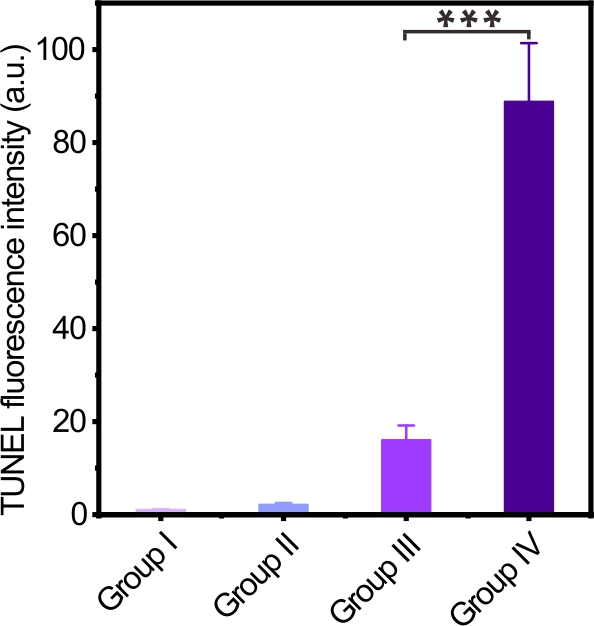


**Fig. S18**: The quantitative analysis of TUNEL fluorescence intensities in each treatment group.


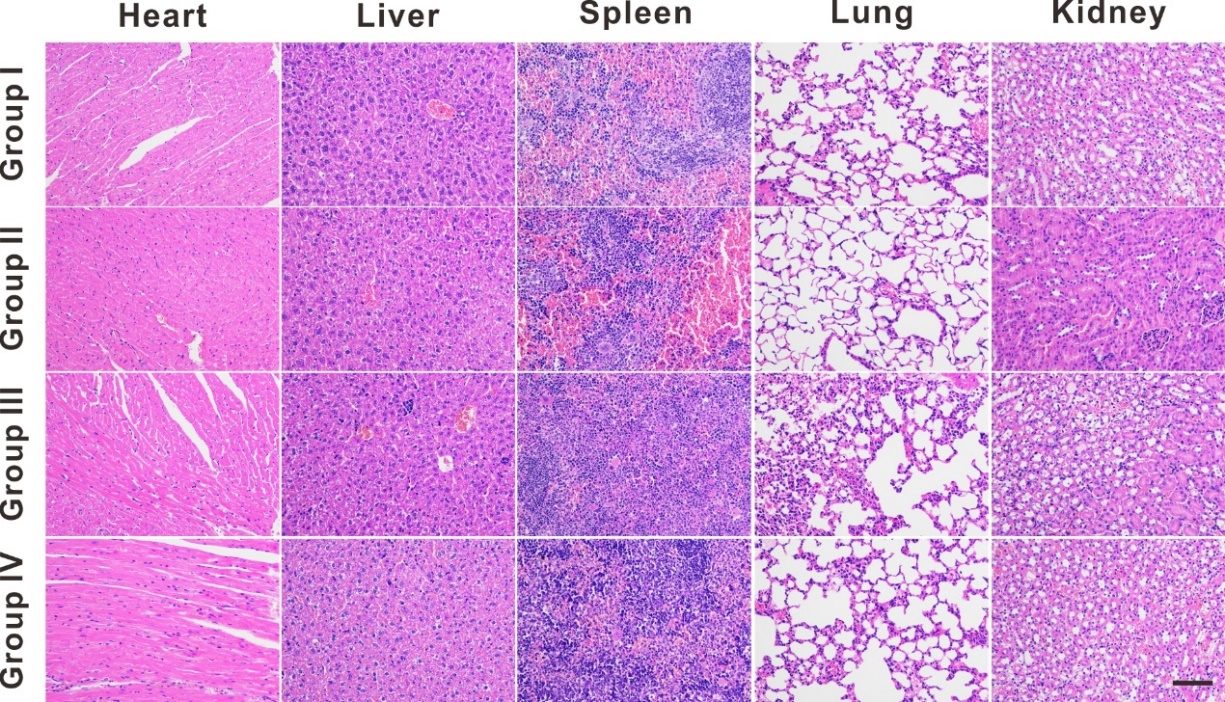


**Fig. S19**: H&E staining images of major health tissues including heart, liver, spleen, lung and kidney of mice in each treatment group (Scale bar = 50 μm).

**
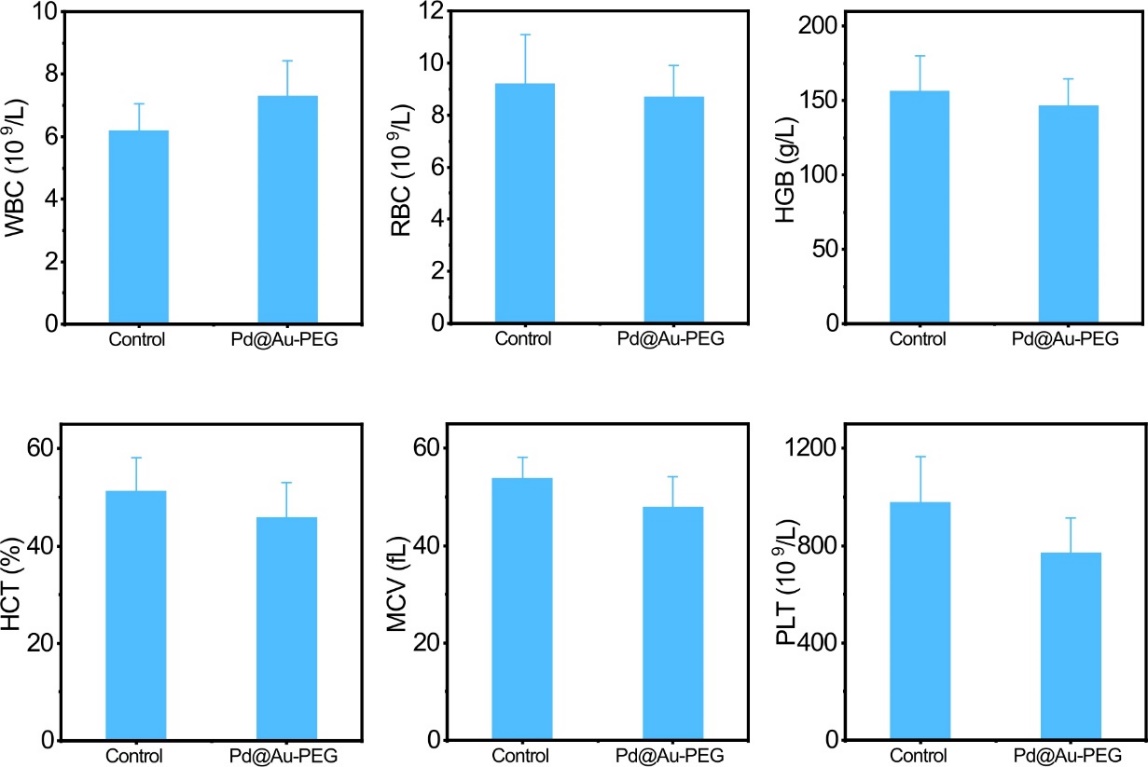
**

**Fig. S20**: Hematological index measurements of Pd@Au-PEG NSs-injected mice.
